# A strategy for optimal fitting of multiplicative and additive hazards regression models

**DOI:** 10.1186/s12874-021-01273-2

**Published:** 2021-05-06

**Authors:** François Lefebvre, Roch Giorgi

**Affiliations:** 1 Aix Marseille Univ, INSERM, IRD, SESSTIM, Marseille, France; 2 Groupe Méthode en Recherche Clinique, Service de Santé Publique, Hôpitaux Universitaires de Strasbourg, Strasbourg, France; 3 Aix Marseille Univ, APHM, INSERM, IRD, SESSTIM, Hop Timone, BioSTIC, Marseille, France

**Keywords:** Multiplicative hazards regression model, Additive hazards regression model, Survival analysis

## Abstract

**Background:**

In survival analysis, data can be modeled using either a multiplicative hazards regression model (such as the Cox model) or an additive hazards regression model (such as Lin’s or Aalen’s model). While several diagnostic tools are available to check the assumptions underpinning each type of model, there is no defined procedure to fit these models optimally. Moreover, the two types of models are rarely combined in survival analysis. Here, we propose a strategy for optimal fitting of multiplicative and additive hazards regression models in survival analysis.

**Methods:**

This section details our proposed strategy for optimal fitting of multiplicative and additive hazards regression models, with a focus on the assumptions underpinning each type of model, the diagnostic tools used to check these assumptions, and the steps followed to fit the data. The proposed strategy draws on classical diagnostic tools (Schoenfeld and martingale residuals) and less common tools (pseudo-observations, martingale residual processes, and Arjas plots).

**Results:**

The proposed strategy is applied to a dataset of patients with myocardial infarction (TRACE data frame). The effects of 5 covariates (age, sex, diabetes, ventricular fibrillation, and clinical heart failure) on the hazard of death are analyzed using multiplicative and additive hazards regression models. The proposed strategy is shown to fit the data optimally.

**Conclusions:**

Survival analysis is improved by using multiplicative and additive hazards regression models together, but specific steps must be followed to fit the data optimally. By providing different measures of the same effect, our proposed strategy allows for better interpretation of the data.

**Supplementary Information:**

The online version contains supplementary material available at 10.1186/s12874-021-01273-2.

## Background

Clinical studies are often aimed at assessing the relationship between explanatory variables and time-to-event outcomes such as survival time. In survival analysis, the presence of censored observations requires the use of specific models. The most commonly used models for this purpose focus directly on the hazard function and can be divided into two types: multiplicative hazards regression models and additive hazards regression models. The most popular multiplicative hazards regression model is the Cox proportional hazards model [1]. In this model, covariates act multiplicatively on the baseline hazard, which is expressed as a time-dependent function without assumptions regarding its shape. The Cox proportional hazards model has two main advantages: it gives a hazard ratio, which allows for interpreting covariate effects as relative risks, and it is easy to compute, which means that it can be applied with practically any statistical software. However, this model is based on two assumptions that must be satisfied to ensure correct fitting of the data, and, consequently, correct interpretation of covariate effects. The first is that covariate effects are constant over time; this rather strong assumption, known as the proportional hazards assumption, results in biased estimates when it is violated [2]. A second assumption, known as the assumption of log-linearity, is that the effects of continuous covariates are log-linear. One typically uses an extended Cox model with time-varying effects when the first assumption is not met [3, 4] and an extended Cox model with non-log-linear effects when the second assumption is not respected [4].

By contrast, in additive hazards regression models, covariates act additively on the baseline hazard. The first additive hazards regression model was proposed by Aalen [5, 6]. In this non-parametric model, covariates effects are modeled as regression functions that can vary over time, which means that the proportional hazards assumption does not apply. Indeed, the only assumption underpinning this model is the linearity of continuous covariates. Aalen’s model gives hazard differences that are then interpreted by plotting cumulative hazards over time. The main advantage of this model is that it allows for investigating the effects of a given covariate over time. Although this model is more flexible than the Cox proportional hazards model, it is less commonly used because it is not well-known and because cumulative hazards are more difficult to interpret than hazard ratios. In case of time-constant effect of the covariates, one can use the additive model proposed by Lin et al. [7], which is a particular case of Aalen’s model. In Lin’s model, regression functions are constant except for the baseline hazard.

While both multiplicative and additive hazards regression models capture the effects of covariates, they allow for interpreting these effects differently. The hazard ratio given by multiplicative models is interpreted as a relative risk. By contrast, the cumulative hazard given by additive models is interpreted as the difference in outcome incidence due to exposure when the cumulative hazard is small (interpretation becomes much more difficult when the cumulative hazard is important) [8]. Accordingly, one can choose either model depending on whether a hazard ratio or a cumulative hazard is preferred as a measure of covariate effects. Generally speaking, additive hazards regression models are more appropriate to determine the effects of exposure in an epidemiologic context [9, 10], and multiplicative hazards regression models are preferred in all other situations. To date, however, the two models have rarely been combined in survival analysis. Moreover, no procedure has been defined to perform optimal fitting of the two models, i.e. which allows obtaining models that fit the data while respecting the assumptions underpinning each type of model.

The aim of this study is to propose a strategy for the optimal fitting of multiplicative and additive hazards regression models in survival analysis.

The structure of the article is as follows. The Methods section begins by detailing our strategy for optimal fitting of multiplicative and additive hazards regression models, with a focus on the assumptions underpinning each type of model, the diagnostic tools used to check these assumptions, and the steps followed to fit the data. The section ends by summarizing the differences between multiplicative and additive hazards regression models. In the Results section, our proposed strategy is applied to a dataset of patients with myocardial infarction (TRACE data frame) to analyze the effects of 5 covariates (age, sex, diabetes, ventricular fibrillation, and clinical heart failure) on the hazard of death. Finally, the Discussion section provides an interpretation of our findings along with concluding remarks.

## Methods

### The proposed strategy

Our strategy for optimal fitting of multiplicative and additive hazards regression models is detailed below. In all equations, *x* is a vector of *k* covariates (${x}_i^T=\left({x}_{i1},\dots, {x}_{ik}\right)$), and *λ*_0_(*t*) is the baseline hazard as a non-parametric function of time. To simplify notation, all the presented covariates are time-independent covariates but all the models and the diagnostic tools can be used, unless otherwise specified, with time-dependent covariates [4, 11].

#### Multiplicative hazards regression model

##### Cox proportional hazards model

The main model in our strategy is the popular Cox proportional hazards model [1]. Two assumptions underpin this model: the proportional hazards assumption and the assumption of log-linearity.

In the Cox proportional hazards model, for a given subject *i*, the hazard is written mathematically as ${\lambda}_i\left(t|{x}_i\right)={\lambda}_0(t){e}^{x_i^T\beta }$, where *β* is the vector of *k* parameters *β* = (*β*_1_, …, *β*_*k*_)^*T*^ measuring the effects of the covariates on the hazard. The parameter *β* is estimated by maximization of the partial likelihood. The exponential of the estimated parameter $\hat{\beta}$, i.e. the hazard ratio, is interpreted as a relative risk.

Thus, for two subjects *i* and *j*, the hazard ratio is constant over time and is written as:

$$ \frac{\lambda_i\left(t|{x}_i\right)}{\lambda_j\left(t|{x}_j\right)}=\frac{\lambda_0(t){e}^{x_i^T\beta }}{\lambda_0(t){e}^{x_j^T\beta }}=\frac{e^{x_i^T\beta }}{e^{x_j^T\beta }}. $$

##### Extended Cox model in case of non-proportional hazards

In our strategy, two diagnostic tools are used to check whether or not the proportional hazards assumption is satisfied. The first is a common test which consists in estimating the correlation between the Schoenfeld residuals and the rank order of event times [12]. The proportional hazards assumption is considered satisfied when the *p*-value is greater than 0.05. The second diagnostic tool is the graphical approach most commonly used to represent the effects of a covariate on the hazard over time. In this approach, the Schoenfeld residuals [13] obtained with a Cox proportional hazards model fitted with each covariate are plotted against the rank order of event times, and a smooth curve is then superimposed on the plot. The obtained curve represents the variation of parameter *β* (i.e. the log-hazard ratio of the covariate effects) over time. The proportional hazards assumption is considered satisfied if the curve is horizontal.

When the proportional hazards assumption is respected for a given covariate, the Cox proportional hazards model is fitted with this covariate.

When the assumption is not satisfied, an extended Cox non-proportional hazards model is fitted with a function of the time-dependent parameter *β*(*t*) [4]. Here, the hazard is written as ${\lambda}_i\left(t|{x}_i\right)={\lambda}_0(t){e}^{x_i^T\beta (t)}$, where *β*(*t*) is defined based on knowledge of the variation or on the results obtained with either of the diagnostic tools above.

The above process is repeated until the proportional hazards assumption is satisfied for each covariate.

##### Extended Cox model in case of non-log-linearity

As noted earlier, the Cox proportional hazards model assumes the log-linearity of continuous covariates. Thus, for a continuous covariate *x* and two subjects *i* and *j*, the hazard ratio is written as $\frac{\lambda_i\left(t|{x}_i\right)}{\lambda_j\left(t|{x}_j\right)}=\frac{\lambda_0(t){e}^{x_i\beta }}{\lambda_0(t){e}^{x_j\beta }}=\frac{e^{x_i\beta }}{e^{x_j\beta }}={e}^{\left({x}_i-{x}_j\right)\beta }$ and depends only on the difference between *x*_*i*_ and *x*_*j*_. For instance, for the continuous covariate “age”, the relative risk between a 25- and a 26-year-old is the same as that between an 80- and an 81-year-old.

In our strategy, the assumption of log-linearity is checked by representing the effects of each continuous covariate on the hazard using the martingale residuals [14]. The martingale residuals are defined as the difference between the observed number of events for an individual (i.e. 1 when there is an event; 0 otherwise) and the number of events estimated with the Cox proportional hazards model. The lowess smooth of the martingale residuals obtained with a null Cox proportional hazards model (i.e. a Cox model with no fitted covariate) is plotted against the continuous covariate, which gives the functional form of this covariate on the hazard [4]. If the obtained curve is straight, then the assumption of log-linearity is satisfied.

When the assumption of log-linearity is satisfied for a given covariate, the Cox proportional hazards model is fitted with the non-transformed covariate.

When this assumption is not satisfied, the best functional form is selected for each covariate using an extended Cox model. In this model, the hazard is written as${\lambda}_i\left(t|{x}_i\right)={\lambda}_0(t){e}^{f\left({x}_i^T\right)\beta }$, where *f*(*x*) is the functional form of covariate *x*. Different functional forms of the continuous covariate are modeled directly using special functions like fractional polynomials or regression splines. Then, the lowess smooth of the martingale residuals obtained with the extended Cox model fitted with these functional forms is plotted against the continuous covariate [15]: the functional forms that give roughly horizontal curves are considered good candidates to satisfy the assumption of log-linearity. The best functional form is then selected using a model selection criterion - for instance, the Akaike information criterion (AIC) for non-nested models.

The above process is repeated until the assumption of log-linearity is met for each continuous covariate.

In case of time-dependent continuous covariate, there is no single value for each subject, so the lowess smooth of the martingale residuals cannot easily be plotted against the continuous covariate [4]. The assumption of log-linearity must be checked with another diagnostic tool presented below.

##### Extended Cox model in case of non-proportional hazards and non-log-linearity

The diagnostic tools used to check the proportional hazards assumption (Schoenfeld residuals) and the assumption of log-linearity (martingale residuals) for a single continuous covariate rely on the other assumption being true. While different methods can be used to check the two assumptions simultaneously (Sasieni and Winnett [16] and Pohar Perme and Andersen [17]), our strategy employs that proposed by Pohar Perme and Andersen [17]. This method is based on pseudo-observations, which were introduced for regression modeling in event-history analysis by Andersen et al. [18].

In survival analysis, for an individual *i*, the pseudo-observation ${\hat{S}}_i(t)$ is defined as the difference between *n* times the survival $\hat{S}(t)$ estimated with the Kaplan-Meier method on the whole sample and *n* minus one times the survival ${\hat{S}}^{-i}(t)$ estimated with the Kaplan-Meier method after leaving out the *i*^th^ individual. One pseudo-observation is thus obtained for each individual at each event time. To check the two assumptions simultaneously, the pseudo-observations are transformed to obtain a linear expression between survival and covariate effects. Thus, for a continuous covariate *z*, the hazard is written as *λ*(*t*| *z*) = *λ*_0_(*t*)*e*^*zβ*^ such that survival is *S*(*t*| *z*) = exp(− ∫ *λ*(*t*| *z*)*dt*) = exp(− ∫ *λ*_0_(*t*)*e*^*zβ*^*dt*) = exp(−*Λ*_0_(*t*)*e*^*zβ*^), where ${\varLambda}_0(t)={\int}_0^t{\lambda}_0(t) dt$. With the cloglog transformation of the smoothed curves of pseudo-observations, we obtain log(− log(*S*(*t*| *z*))) = log(*Λ*_0_(*t*)) + *zβ*. The effects of the covariate on survival are represented by plotting the cloglog-transformed smoothed pseudo-observations against the continuous covariate. Because covariate effects can vary over time, pseudo-observations are usually plotted at selected time points (e.g. 9 curves corresponding to 9 deciles of the event times distribution). The two assumptions are satisfied if the obtained curves are parallel straight lines with the same slope *β*.

When the two assumptions are satisfied for a given covariate, the Cox proportional hazards model is fitted with the non-transformed covariate.

When neither of the assumptions is satisfied, an extended Cox model with time-varying effects and non-log-linear effects is used. In this model, for a continuous covariate *z*, the hazard is written as ${\lambda}_i\left(t|{z}_i\right)={\lambda}_0(t){e}^{f\left({z}_i\right)\beta (t)}$. The model is fitted with functions of the time-dependent parameter and with functional forms of the continuous covariate.

The above process is repeated until both the proportional hazards assumption and the assumption of log-linearity are satisfied for each continuous covariate.

##### Goodness-of-fit assessment using Arjas plots

The goodness-of-fit of the multiplicative hazards regression model fitted with each covariate is assessed using the method proposed by Arjas [19]. This method known as Arjas plots consists in plotting the observed number of patients with the event against the number of patients with the event estimated with the model. If the observed number and the estimated number of patients with the event are close and the obtained curve roughly matches the diagonal line, then the model has goodness-of-fit. Note that in the case of continuous covariates, groups of individuals are defined by dividing the continuous covariate distribution into several strata (e.g. 4 strata according to the quartiles of the continuous covariate distribution).

The model has no goodness-of-fit when the curves systematically deviate from the diagonal for some groups of individuals, indicating an excess or a lack of predicted events. When this occurs, the proportional hazards assumption and the assumption of log-linearity must be checked again for each covariate (as described above) until goodness-of-fit is achieved.

##### Multivariate multiplicative model

The proportional hazards assumption is checked for the multiplicative model fitted with all covariates by testing the correlation between the Schoenfeld residuals and the rank order of event times. The goodness-of-fit of the multivariate model is then assessed using Arjas plots. The entire process is repeated until the proportional hazards assumption is satisfied for all covariates and the multivariate model has goodness-of-fit.

##### Step-by-step strategy for optimal fitting of multiplicative hazards regression models

To summarize, in order to optimally fit a multiplicative hazards regression model in survival analysis, the following step-by-step strategy is implemented:

1. Check the assumption of log-linearity for each continuous covariate using the martingale residuals (or the plot of the cloglog-transformed smoothed pseudo-observations against the time-dependent continuous covariate). In case on non-log-linearity, select the best functional form of the continuous covariate using an extended Cox model. Repeat this step until the assumption of log-linearity is satisfied for each continuous covariate.
2. Check the proportional hazards assumption for each covariate by testing the correlation between the Schoenfeld residuals and the rank order of event times. In case of non-proportional hazards, model a function of the time-varying parameter in an extended Cox model. Repeat this step until the proportional hazards assumption is satisfied for each covariate.
3. Check simultaneously the proportional hazards assumption and the assumption of log-linearity by plotting the cloglog-transformed smoothed pseudo-observations against each continuous covariate. In case of non-log-linearity and non-proportional hazards, use an extended Cox model. Repeat steps 1, 2, and 3 until both assumptions are satisfied for each continuous covariate.
4. Assess the goodness-of-fit of the multiplicative hazards regression model for each covariate using Arjas plots. Repeat steps 1, 2, 3, and 4 until the model has goodness-of-fit for each covariate.
5. Check the proportional hazards assumption for the multiplicative model fitted with all covariates using the same procedures as in Step 2. Repeat steps 1, 2, 3, 4, and 5 until the multivariate model has goodness-of-fit.

#### Additive hazards regression model

##### Aalen’s model

The additive hazards regression model proposed by Aalen is used to estimate the additive effects of a covariate on the baseline hazard [5, 6]. In this model, for a given subject *i*, the hazard is written as ${\lambda}_i\left(t|{x}_i\right)={\lambda}_0(t)+{x}_i^T\alpha (t)$, where *α*(*t*) is the vector of *k* non-parametric regression functions *α*(*t*) = (*α*_1_(*t*), …, *α*_*k*_(*t*))^*T *^measuring the effects of the covariates on the hazard. The non-parametric regression function *α*(*t*) is estimated by the least squares method at each event time for at-risk individuals only [5, 6]. The only assumption of this model is that continuous covariates are linear. The diagnostic tools used to check this assumption are presented below.

##### Lin’s model

Lin’s model [7] is a particular case of Aalen’s model, in which all regression functions except the baseline hazard are constant over time. Thus, for a given subject *i,* the hazard is written as ${\lambda}_i\left(t|{x}_i\right)={\lambda}_0(t)+{x}_i^T\gamma$, where *γ* is the vector of *k* parameters *γ* = (*γ*_1_, …, *γ*_*k*_)^*T *^measuring the effects of covariates on the hazard. As in the Cox proportional hazards model, the parameter *γ* is estimated by maximization of the partial likelihood.

In addition to assuming the linearity of continuous covariates, Lin’s model assumes that covariate effects are constant over time. The assumption of constant effects is checked for each covariate by plotting the cumulative hazards estimated with Lin’s and Aalen’s models against time. The assumption is satisfied if the obtained curve has a constant slope. If the effects of all covariates are constant over time, Lin’s model is used; otherwise, Aalen’s model is used.

##### Extended Aalen’s model in case of non-linearity

Here, the assumption of linearity of continuous variables is checked not with the martingale residuals as in the extended Cox model, but with the pseudo-observations. Specifically, Lin’s model (based on the assumptions of linearity and constant effects) is used to model the functional form of the continuous covariate after a transformation of the pseudo-observations. Thus, for a continuous covariate *z,* the hazard is written as *λ*(*t*| *z*) = *λ*_0_(*t*) + *γz* such that survival is *S*(*t*| *z*) = exp(− ∫ *λ*(*t*| *z*)*dt*) = exp(− ∫ *λ*_0_(*t*) + *γzdt*) = exp(−*Λ*_0_(*t*) − *γzt*), where ${\varLambda}_0(t)={\int}_0^t{\lambda}_0(t) dt$. After log-transformation of the pseudo-observations, we obtain $\frac{-\log \left(S\left(t|z\right)\right)}{t}=\frac{\varLambda_0(t)}{t}+\gamma z$. The effect of the continuous covariate on survival is represented by plotting the log-transformed smoothed pseudo-observations against the covariate, and then superimposing smooth curves on the scatter plot. Note that because covariate effects can vary over time, the pseudo-observations are usually plotted at selected time points.

The shape of the obtained curves gives the functional form of the continuous covariate and indicates which model to use. If the obtained curves are straight lines, then the assumption of linearity is respected: in that case, Aalen’s model or Lin’s model is used (depending on whether or not covariate effects are constant, as explained above). If the obtained curves are not straight lines and are not parallel, then neither the assumption of linearity nor the assumption of constant effects is satisfied: in that case, an extended Aalen’s model is used (see below). Finally, if the obtained curves are not straight lines and are parallel (with the same slope *γ*), then the assumption of linearity is not satisfied but the assumption of constant effects is respected: in that case, an extended Lin’s model is used (see below).

In the extended Aalen’s model, the functional form of the continuous covariate is modeled directly using special functions like fractional polynomials or regression splines. Here, the hazard is written as${\lambda}_i\left(t|{x}_i\right)={\lambda}_0(t)+f\left({x}_i^T\right)\alpha (t)$, where *f*(*x*) is the functional form of the covariate *x*. To determine whether the functional form of the continuous covariate is appropriate, the variation of the martingale residual processes over time is assessed graphically [6]. For a given group of individuals, the cumulative sum of the martingale residual processes is the difference between the observed number of events and the number of events estimated with the model fitted for this group.

First, groups of individuals are defined by dividing the continuous covariate distribution into several strata (e.g. 4 strata according to the quartiles of the continuous covariate distribution). Then, the cumulative sum of the martingale residual processes for each group is plotted against time with their confidence bounds. When the fit is correct, the obtained curves are close to the horizontal axis and their confidence bounds do not cross the horizontal axis. Finally, chi-square tests are performed at the end of patient follow-up to compare the observed number of events to the estimated number of events (for each stratum and for all strata) [6]. If the obtained *p*-value is statistically significant, the functional form is rejected and another functional form is tested.

The above process is repeated until the assumption of linearity is met for each continuous covariate.

##### Extended Lin’s model in case of non-linearity

As noted above, when the continuous covariates are non-linear and the covariate effects are constant over time, an extended Lin’s model is used. In this model, the hazard is written as${\lambda}_i\left(t|{x}_i\right)={\lambda}_0(t)+f\left({x}_i^T\right)\gamma$ where *f*(*x*) is the functional form of the covariate *x*. Note that the functional form of the continuous covariate is modeled directly using special functions like fractional polynomials or regression splines.

##### Goodness-of-fit assessment using Arjas plots

The goodness-of-fit of the additive hazards regression model is assessed using Arjas plots [5]. Note that this procedure is not necessary in the case of categorical covariates because in Aalen’s model the observed number of events is always equal to the estimated number of events at all time points.

##### Multivariate additive model

The assumption of linearity is checked for the additive model fitted with all covariates by assessing the variation of the martingale residual processes over time. Then, the goodness-of-fit of the multivariate model is assessed using Arjas plots. The entire process is repeated until the assumption of linearity is satisfied for all covariates and the multivariate model has goodness-of-fit.

##### Step-by-step strategy for optimal fitting of additive hazards regression models

To summarize, in order to optimally fit an additive hazards regression model in survival analysis, the following step-by-step strategy is implemented:

1. Check the assumption of linearity for each covariate using log-transformed smoothed pseudo-observations. In case on non-linearity, select the best functional form of the continuous covariate by assessing the variation of the martingale residual processes over time. Repeat this step until the assumption of linearity is satisfied for each continuous covariate.
2. If the assumption of linearity is satisfied, check the assumption of constant effects for each covariate by plotting the cumulative hazards estimated with Lin’s and Aalen’s models against time. If the effects of all covariates are constant, use Lin’s model; otherwise, use Aalen’s model.
3. Assess the goodness-of-fit of the additive hazards regression model for each continuous covariate using Arjas plots. Repeat steps 1, 2, and 3 until the model has goodness-of-fit for each continuous covariate.
4. Check the assumption of linearity for the additive model fitted with all the covariates using the same procedure as in step 1. Repeat this step with another functional form of the continuous covariate until the assumption of linearity is satisfied for all covariates.

#### Differences between multiplicative and additive hazards regression models

In the Cox proportional hazards model, for a binary covariate *z*, the hazard is written as *λ*(*t*| *z*) = *λ*_0_(*t*)*e*^*zβ*^. The effect of the covariate on the hazard is measured by the hazard ratio, which is written as $HR={e}^{\beta }=\frac{\lambda \left(t|z=1\right)}{\lambda \left(t|z=0\right)}=\frac{\lambda \left(t|z=1\right)}{\lambda_0(t)}$. The importance of the effect of the covariate on the hazard depends on the baseline hazard: when the baseline hazard is very low, the hazard remains low for the exposed subject even if the hazard ratio is important. Moreover, the effect of the covariate is interpreted using a single parameter - the hazard ratio - which is constant over time. In the case of the extended Cox model with time-varying effects, the hazard ratio varies over time, and so the interpretation requires plotting hazard ratios against time.

In Aalen’s model, for the same covariate *z*, the hazard is written as *λ*(*t*| *z*) = *λ*_0_(*t*) + *α*(*t*)*z*. Here, the effect of the covariate is measured by the cumulative hazard, which is written as *α*(*t*) = *λ*(*t*| *z* = 1) − *λ*(*t*| *z* = 0). The cumulative hazard highlights the importance of the effect of exposure over time, whatever the baseline hazard. It represents the attributable fraction due to exposure, hence its common use in epidemiology. Since the cumulative hazard is a function, it can be plotted to show the evolution of the effect over time.

In short, each type of model allows for a different interpretation of the effect of the covariate on the hazard.

#### Estimating procedures

Estimating procedures for multiplicative and additive hazards regression models are available in the major statistical software (SAS, STATA, and R). At the moment, however, some of the diagnostic tools used in our strategy are only available in R. All of our analyses were therefore performed using R software (script provided in Additional file 1).

## Results

### Motivating example

In this section, our proposed strategy for optimal fitting of multiplicative and additive hazards regression models is applied to a motivating example: the TRACE data frame provided in the timereg R package. This data frame is a subset of a dataset extracted from a study of 4259 patients with myocardial infarction who were admitted to a hospital in Denmark in 1977–1988 and were followed until death or censoring [20].

The 1878 patients included in the TRACE data frame had a mean age of 67.0 years (sd: 11.4). Overall, 52.29% of patients had clinical heart failure, 69.54% of patients were women, 10.01% of patients had diabetes, and 7.24% had ventricular fibrillation. Median survival was 6.52 years [6.09; 7.25]. The TRACE data frame is interesting for our purposes because it contains covariates with well-known time-varying effects on the hazard of death (ventricular fibrillation) as well as covariates with constant effects on the hazard of death (diabetes and sex) [21].

Our proposed strategy is used to investigate the hazard of death in the included group of patients with myocardial infarction. The effects of age (continuous covariate named age), clinical heart failure (binary covariate named chf), sex (binary covariate named sex), diabetes (binary covariate named dia), and ventricular fibrillation (binary covariate named vf) on the hazard of death are examined below. Note that because Aalen’s model assumes no ties between event times, a random number has been added to each survival time such that no two survival times are identical.

### Application of the proposed strategy for optimal fitting of multiplicative hazards regression models

#### Checking the assumption of log-linearity

**Fig. 1:**
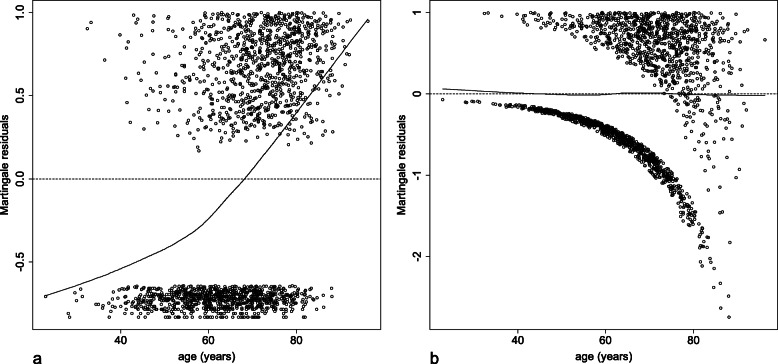
Martingale residuals plotted against the continuous covariate age with **a** a null Cox proportional hazards model and with **b** a Cox proportional hazards model fitted with the exponential of age/100. A lowess smooth curve is superimposed on each plot (solid line)

To select the best functional form of the continuous covariate age, the martingale residuals obtained with a null Cox proportional hazards model are plotted against age. As shown in Fig. 1a, the curve representing the effect of age is not a straight line, indicating a deviation from log-linearity. Specifically, there is an increase in the slope of the curve, which suggests an exponential function and perhaps even a quadratic function.

After dividing age by 100 to avoid too large values, a Cox proportional hazards model is fitted with an exponential of age/100. Figure 1b shows the plot of the martingale residuals. The smooth curve is a horizontal straight line, indicating that the exponential of age/100 is an appropriate functional form.

A Cox proportional hazards model is then fitted with a quadratic effect of age/100. The smooth curve obtained after plotting the martingale residuals (Figure not shown) is also a horizontal straight line.

The AIC of the two models is calculated to determine which of the last two functional forms is more appropriate. The AIC of the Cox proportional hazards model fitted with the quadratic effect of age/100 (13,533.73) is greater than that of the Cox proportional hazards model fitted with the exponential of age/100 (13,531.83). The latter functional form is selected.

#### Checking the proportional hazards assumption

The proportional hazards assumption is checked for each of the 5 covariates by testing the correlation between the Schoenfeld residuals and the rank order of event times (Table 1). For the covariates chf and vf, the assumption of proportionality is not satisfied (*p*-values < 0.05), indicating that an extended Cox model with time-varying effects is needed. By contrast, the effects of the exponential of age/100, sex, and dia do not vary statistically over time, and so the effect of each variable is modeled as constant over time.

**Table 1:** *P*-values of the correlation between the Schoenfeld residuals and the rank order of event times for the 5 predictive covariates

Covariate	*P*-value
exp (age/100)	0.29
sex	0.66
chf	1.8 × 10^−4^
dia	0.23
vf	9.5 × 10^−10^

**Fig. 2:**
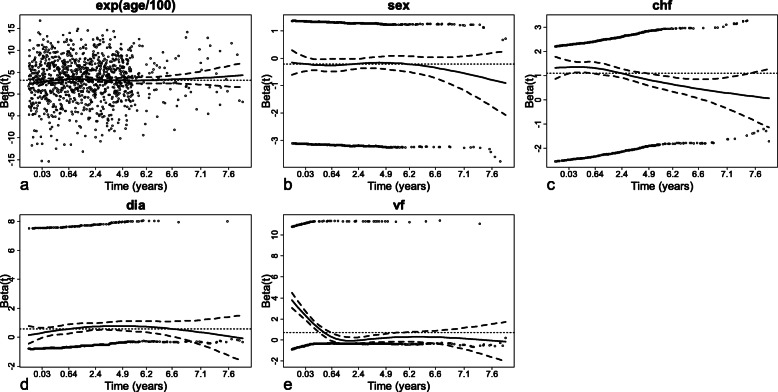
Schoenfeld residuals plotted against the rank order of event times for **a** the exponential of age/100, **b** sex, **c** chf, **d** dia, and **e** vf. A lowess smooth (solid line) corresponding to the parameter β with its 95% confidence intervals (dashed lines) is superimposed on each plot

Figure 2 shows the plot of the Schoenfeld residuals for each of the 5 predictors. The smooth curves are roughly horizontal for the exponential of age/100, sex, and dia, indicating that these covariates have constant effects over time. Accordingly, a Cox proportional hazards model is used for these three covariates.

For the covariate chf, the smooth curve decreases with a roughly constant slope, indicating a linear time-dependent effect. The covariate chf is thus modeled as follows: $\lambda \left(t| chf\right)={\lambda}_0(t){e}^{chf\times \left({\beta}_{chf}+{\beta}_{chf t}\times t\right)}$. The Schoenfeld residuals test indicates that the assumption of proportional hazards is satisfied for the parameters *β*_*chf*_ and *β*_*chft*_ (*p*-values of 0.85 and 0.41, respectively).

For the covariate vf, the smooth curve decreases with a roughly constant slope until 0.64, and then remains horizontal afterwards. Accordingly, a simple model with a break of slope accounting for the two variations of the hazard is used. To choose the best cut-off time for the break of slope, different cut-off times ranging from 0 to 2.4 (in intervals of 0.01) are assessed using the AIC. The minimal AIC obtained is 13,824.78 for a cut-off time of 0.15. The covariate vf is therefore modeled as follows: $\lambda \left(t| vf\right)={\lambda}_0(t){e}^{vf\times \left({\beta}_{vf}+{\beta}_{vf t}\times t+{\beta}_{vf t2}\times \left(t-0.15\right)I\left(t>0.15\right)\right)}$. The Schoenfeld residuals test shows that the proportional hazards assumption is satisfied for the parameters *β*_*vf*_, *β*_*vft*_, and *β*_*vft*2_ (*p*-values of 0.60, 0.66, and 0.66, respectively).

#### Assessing goodness-of-fit

**Fig. 3:**
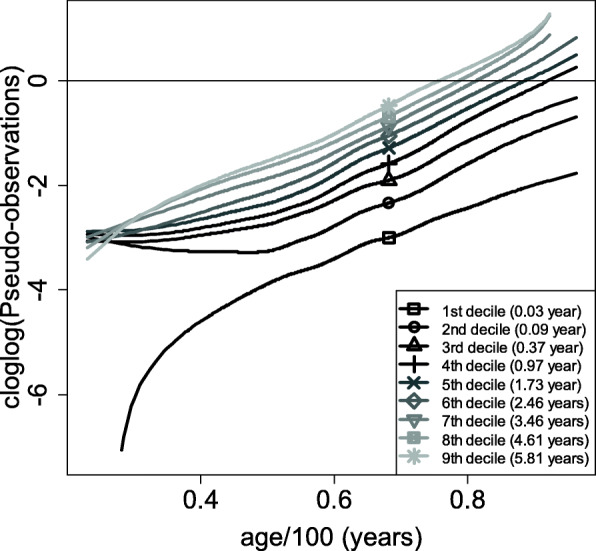
cloglog-transformed smoothed pseudo-observations against age/100 for the 9 deciles of the event times distribution (in years)

Figure 3 shows the plot of the cloglog-transformed smoothed pseudo-observations (cloglog transformation being necessary to check the two assumptions of the Cox model, as shown in the Methods section) against the continuous covariate age/100 for the 9 deciles of the event times distribution (in years). The curves are not entirely straight, which means that the effect is not fully linear. This is consistent with what was found using the martingale residuals (Fig. 1). Furthermore, the curves are not entirely parallel (especially before age/100 = 0.4, i.e. 40 years), indicating that a slightly different functional form of the covariate is needed. The 5 curves representing the 2nd to the 6th deciles are roughly horizontal until 40 years, and then increase afterwards. This indicates an absence of effect of age until 40 years, followed by an almost exponential increase of this effect. Based on the study of the pseudo-observations, the exponential of age/100 is selected.

**Fig. 4:**
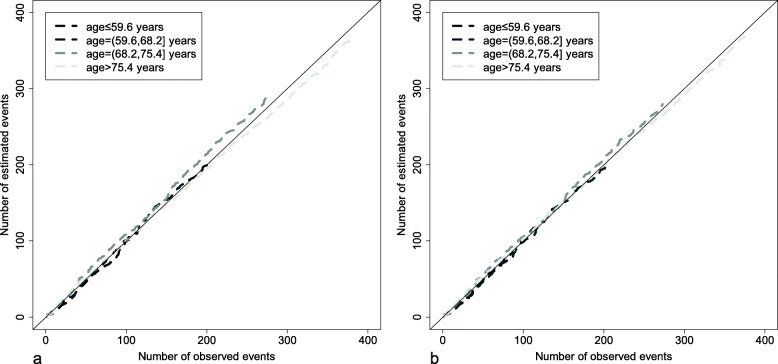
Arjas plots for the Cox proportional hazards model fitted with **a** the linear effect of age and **b** the exponential effect of age/100

Figure 4 shows the Arjas plots for the Cox proportional hazards model fitted with the continuous covariate age in 4 strata defined according to the quartiles of age distribution. The Arjas plot corresponding to the linear effect of age (Fig. 4a) and the Arjas plot corresponding to the exponential effect of age/100 (Fig. 4b) are not very different, but nevertheless suggest that goodness-of-fit is higher for the exponential effect of age/100 than for the linear effect of age (as can already be seen in Fig. 3).

**Fig. 5:**
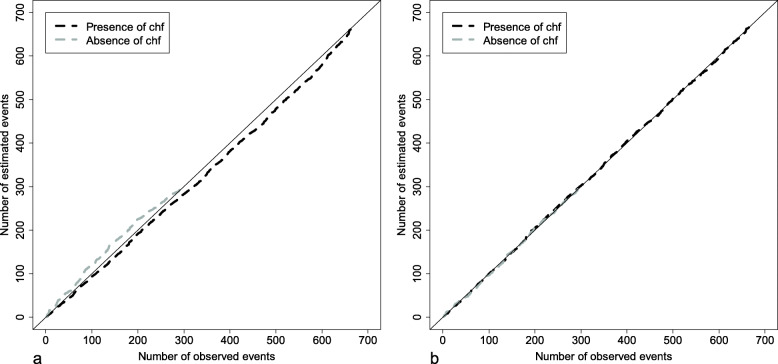
Arjas plots for the Cox proportional hazards model fitted **a** without time-varying effect of chf and **b** with a linear time-varying effect of chf

Figure 5 shows the Arjas plots for the Cox proportional hazards model fitted with the binary covariate chf. In Fig. 5a, the binary covariate chf is modeled without time-dependent effect. The number of estimated events is more important than the number of observed events for patients without clinical heart failure, and it is less important than the number of observed events for patients with clinical heart failure. This indicates that modeling the binary covariate chf without time-varying effect is inappropriate. In Fig. 5b, the binary covariate chf is modeled with the linear time-varying effect obtained earlier (see *Checking the proportional hazards assumption* section). The number of estimated events and the number of observed events are roughly equal for patients with or without clinical heart failure, indicating that modeling the binary covariate chf with the linear time-varying effect is appropriate.

#### Fitting the multivariate multiplicative model

Once the proportional hazards assumption has been checked for each continuous covariate, it is checked for the multivariate model fitted with all the covariates using the same procedure as in step 2 of the univariate analysis. The Schoenfeld residuals test is statistically non-significant for all the covariates except for dia (*p*-value = 0.016). The plot of the correlation between the Schoenfeld residuals and the rank order of event times (Figure not shown) indicates that a linear time-dependent effect of dia is needed. A linear time-dependent effect is added, and so the multivariate model is written as $\lambda (t)={\lambda}_0(t){e}^{\beta_{age}\times \exp \left(\frac{age}{100}\right)+{\beta}_{sex}\times sex+\left({\beta}_{chf}+{\beta}_{chf t}\times t\right) chf+\left({\beta}_{dia}+{\beta}_{dia t}\times t\right) dia+\left({\beta}_{vf}+{\beta}_{vf t}\times t+{\beta}_{vf t2}\times \left(t-0.15\right)I\left(t>0.15\right)\right) vf}$. The Schoenfeld residuals test shows that all the covariates are statistically non-significant with this model.

**Fig. 6:**
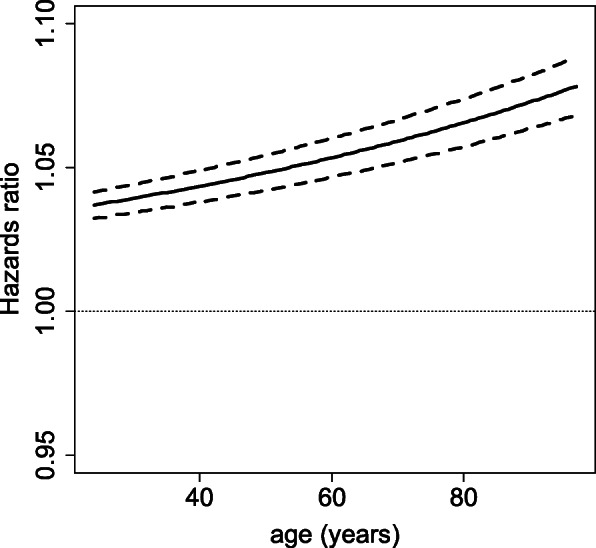
Adjusted hazard ratio (solid line) with its 95% confidence interval (dashed lines) against an increase in 1 year of age

In addition, each parameter is shown to be statistically significant with this model (Table 2). The effects of the covariates exponential of age/100 and sex are constant over time, while the effects of the covariates chf, dia, and vf vary over time. The hazard ratio for sex is 1.20 [1.05; 1.38] in women. As the effect of the exponential of age/100 is log-linear, the effect of age is not log-linear and is therefore interpreted graphically. As expected, the hazard ratio for an increase of 1 year increases with age, i.e. the hazard ratio for 1 year of ageing is more important in older patients than in younger patients (Fig. 6).

**Table 2:** Parameters $\hat{\beta}$ and adjusted hazard ratios with their 95% confidence intervals (CI) for 9 predictive covariates with the multivariate Cox model

Covariate	$\hat{\beta}$	Adjusted Hazard Ratio with 95% CI	*P*-value
exp (age/100)	2.87	17.56 [12.35; 24.97]	< 2 × 10^− 16^
sex	0.18	1.20 [1.05; 1.38]	0.00982
chf	0.95	2.57 [2.06; 3.22]	< 2 × 10^−16^
chft	−0.08	0.92 [0.86; 0.98]	0.01024
dia	0.35	1.42 [1.09; 1.84]	0.00889
diat	0.09	1.10 [1.01; 1.19]	0.03137
vf	2.24	9.39 [6.32; 13.94]	< 2 × 10^−16^
vft	−13.52	1.35 × 10^−6^ [1.03 × 10^−8^; 1.77 × 10^−4^]	5.61 × 10^− 8^
vft2	13.49	7.22 × 10^5^ [4.93 × 10^3^; 1.06 × 10^8^]	1.15 × 10^−7^

**Fig. 7:**
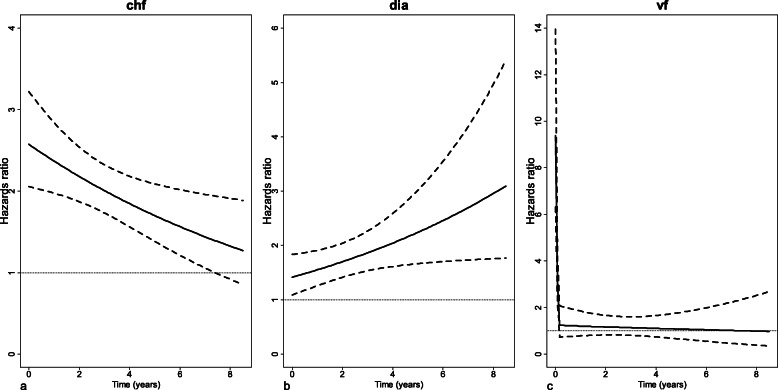
Adjusted hazard ratios (solid lines) with their 95% confidence intervals (dashed lines) against time for the binary covariates chf, dia, and vf

The hazard ratios for the three other binary covariates are not constant but vary over time. They are therefore represented graphically to give a clear idea of their evolution over time (Fig. 7). The hazard ratio for chf decreases linearly over time, and then becomes non-significant at the end of patient follow-up (Fig. 7a). By contrast, the hazard ratio for dia increases linearly, and then becomes important at the end of follow-up (Fig. 7b). Finally, the hazard ratio for vf is very important at first, and then decreases rapidly to become non-significant after 2 months (Fig. 7c).

### Application of the proposed strategy for optimal fitting of additive hazards regression models

#### Checking the assumption of linearity

**Fig. 8:**
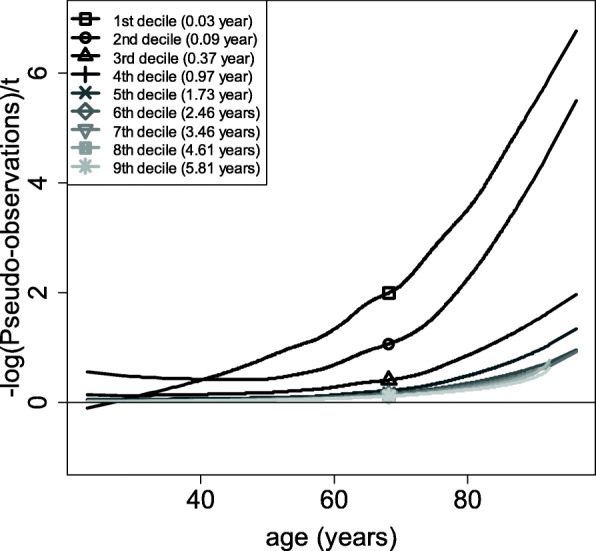
Log-transformed smoothed pseudo-observations against the continuous covariate age for 9 deciles of the event time distribution

**Fig. 9:**
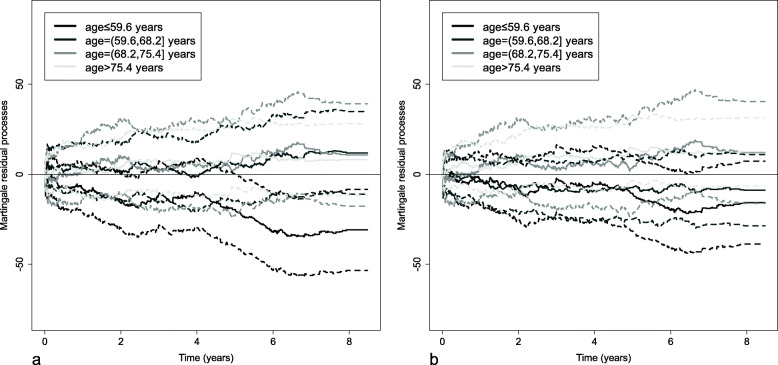
Martingale residual processes (solid lines) with their confidence bounds (dashed lines) in 4 strata defined according to the quartiles of age distribution obtained with an extended Aalen’s model fitted with **a** the exponential of age/10 and **b** the exponential of age/10 with a cut-off at 70 years

To define the functional form of the continuous covariate age, the log-transformed smoothed pseudo-observations (log transformation being necessary to check the assumption of linearity of the additive hazards regression model, as shown in the Methods section) are plotted against age for 9 deciles of the event times distribution. As Fig. 8 indicates, the curves are not straight lines, which means that the effect is not linear, but are nearly parallel, which means that the effect is constant over time. The 7 curves representing the 3rd to the 9th deciles are very similar: they are roughly horizontal until 60 years, and then increase afterwards. This indicates a slight effect of age until 60 years, and then an exponential increase in the effect of age. For the other two deciles, the increase is more important, and also corresponds to an exponential effect. As the exponential of age can take very large values, this covariate is initially divided by ten. Then, to check whether the exponential functional form is appropriate, the martingale residual processes are plotted against time in 4 strata defined according to the quartiles of age distribution (Fig. 9a). The plot indicates that the observed number of younger patients is less important than the estimated number of younger patients at the end of patient follow-up. The results of the chi-square test of the martingale residual processes confirm that modeling with the exponential of age/10 is inappropriate (Table 3). Given that the increase in the slope begins roughly at 70 years, a more flexible functional form is needed to properly account for the increase in the effect of age. The covariate age is therefore modeled as the exponential of age/10 with a cut-off at 70 years. Figure 9b shows that when age is modeled in this way, the martingale residual processes do not significantly deviate from the horizontal axis (for the 4 strata). Likewise, Table 3 indicates that the extended Aalen’s model has goodness-of-fit with this functional form.

**Table 3:** Results of the chi-square tests of the martingale residual processes for the continuous covariate age entered in two extended Aalen’s models

Age	*P*-value	
	exp (age/10)	exp (age/10) + exp (age/10–7)(age > 70)
≤59.6	0.007	0.179
(59.6–68.2]	0.310	0.381
(68.2–75.4]	0.459	0.404
>75.4	0.416	0.197
global	0.056	0.221

#### Checking the assumption of constant effects

**Fig. 10:**
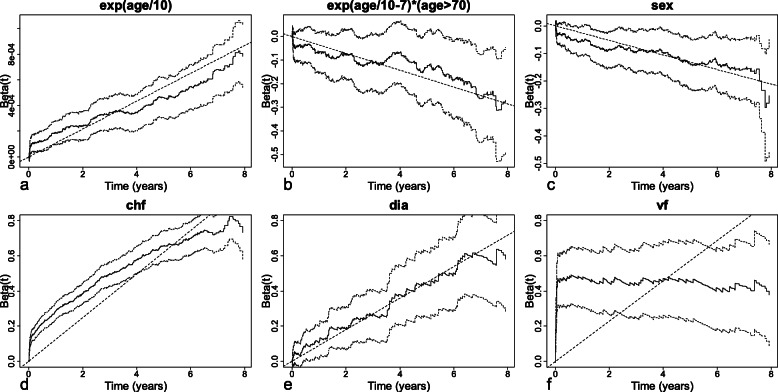
Cumulative hazards with their 95% confidence intervals (dotted lines) estimated with Aalen’s model for 6 covariates. The dashed straight lines represent the cumulative hazards estimated with Lin’s model

The assumption of constant effects is checked for 6 covariates by plotting the cumulative hazards estimated with Lin’s and Aalen’s models. Figure 10 shows that for the exponential of age/10, the exponential of (age-70) × (age > 70), sex, and dia, the cumulative hazards estimated with Lin’s model (dashed straight lines) do not cross the 95% confidence intervals of the cumulative hazards estimated with Aalen’s model (dotted lines). This indicates that the effect of each covariate is constant. For chf, the cumulative hazard increases more quickly at the beginning, and then more slowly afterwards. For vf, the hazard increases considerably until 0.1, and then remains constant afterwards. Since the effect varies over time for some of the covariates, the extended Lin’s model is not appropriate, and the analyses are performed using the extended Aalen’s model.

For the covariates exponential of (age-70) × (age > 70) and sex, the estimated cumulative hazards are negative. This is not an unusual finding in additive hazards regression models.

#### Assessing goodness-of-fit

**Fig. 11:**
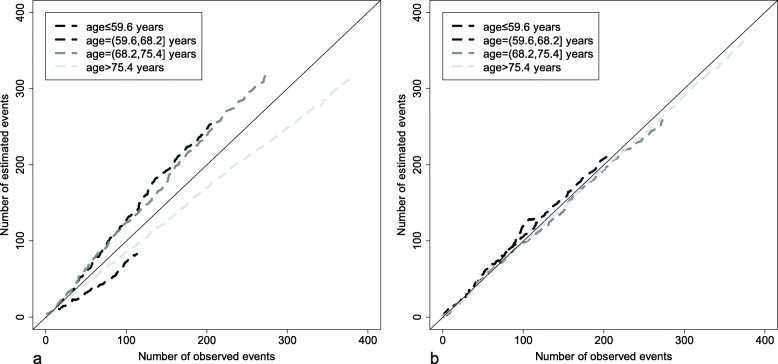
Arjas plots for Aalen’s models fitted with **a** age without transformation and **b** the exponential of age/10 with a cut-off at 70

Figure 11 shows the Arjas plots for Aalen’s model fitted with the continuous covariate age, with 4 strata defined according to the quartiles of age distribution. In Fig. 11a, Aalen’s model is fitted with the continuous covariate age without transformation; in Fig. 11b, Aalen’s model is fitted with the exponential of age/10 with a cut-off at 70 (Fig. 11b). Figure 11a shows an important discrepancy between the number of observed events and the number of estimated events in the 4 strata, indicating a lack of fit when the continuous covariate age is modeled without transformation. By contrast, in Fig. 11b, the number of observed events is close to the number of estimated events. Accordingly, the continuous covariate age is modeled as the exponential of age/10 with a cut-off at 70.

#### Fitting the multivariate additive model

Once the assumption of linearity has been checked for each continuous covariate, it is checked for the multivariate model fitted with all the covariates using the same procedure as in step 1 of the univariate analysis. The test of the martingale residual processes (Table 4) shows no deviation when the continuous covariate age is modeled as an exponential function with a cut-off at 70 (Figure not shown).

**Table 4:** Results of the chi-square tests of the martingale residual processes for the continuous covariate age with in an extended Aalen’s model

Age	*P*-value
≤59.6	0.542
(59.6–68.2]	0.481
(68.2–75.4]	0.756
>75.4	0.316
global	0.589

**Fig. 12:**
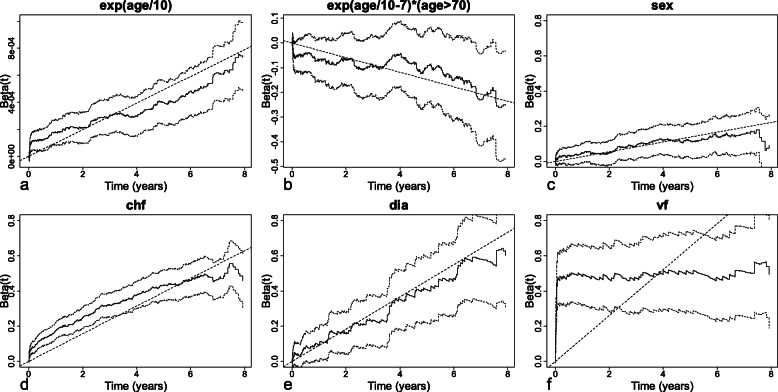
Cumulative hazards with their 95% confidence intervals (dotted lines) estimated with the multivariate Aalen’s model for 6 covariates. The dashed lines represent the cumulative hazards estimated with the multivariate Lin’s model

As shown in Fig. 12, the cumulative regression functions estimated with the multivariate Lin’s and Aalen’s models are significantly different from 0 for all the covariates, indicating a significant effect of these covariates on mortality.

**Fig. 13:**
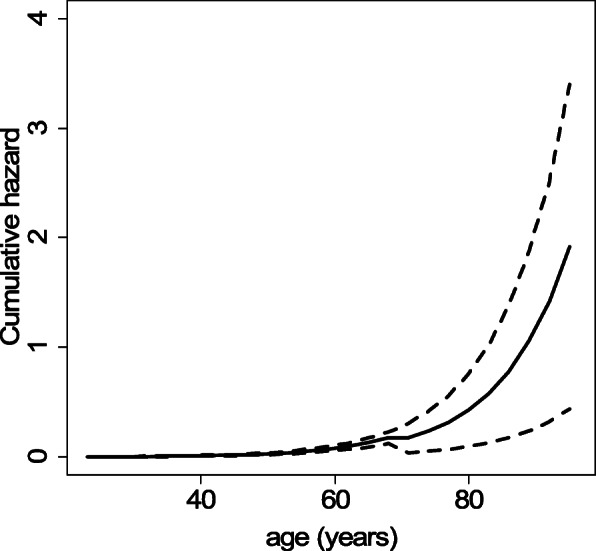
Cumulative hazards with their 95% confidence bounds (dotted lines) estimated with the multivariate Lin’s model for the continuous covariate age at 1 year

The slope of the estimated cumulative regression function is positive for all the binary covariates. The effect is constant for sex and dia but is more important for dia than for sex, indicating that the excess risk of death due to diabetes is more important than the excess risk of death due to sex. For chf, the slope of the estimated cumulative regression function decreases slowly over time, indicating a decrease in the effect of clinical heart failure on mortality. For vf, the slope is very high until 0.1 year, and then becomes horizontal. This indicates that the excess risk of death due to ventricular fibrillation is present during the first 0.1 year of patient follow-up, and then vanishes afterwards. Finally, for the exponential of age/10, the slope of the estimated cumulative regression function is constant and positive until cut-off and then constant and negative afterwards, indicating an absence of variation of the effect over time. However, while the excess risk of death increases exponentially with ageing until 70 years – as shown by the exponential functional form – this increase is less important after 70 years – as shown by the negative slope (Figs. 12 and 13).

## Discussion

This work proposes a strategy for optimal fitting of multiplicative and additive hazards regression models in survival analysis. The proposed strategy has been shown to fit the data optimally. Several points can be made in this regard.

The first point is that several factors condition the choice between multiplicative and additive hazards regression models in survival analysis. The most important factor is knowledge of the effect of the covariate on the hazard of death. If this effect is known to be multiplicative or additive, then the corresponding model (multiplicative hazards regression model or additive hazards regression model, respectively) is used [22, 23]. However, the effect of the covariate on the hazard of death is rarely known in advance. Consequently, the choice between the two types of models is usually made based on whether one wants to obtain a relative risk – e.g. the hazard ratio – or an absolute risk – e.g. the cumulative hazard – as a measure of the effect of a covariate on mortality. Thus, the hazard ratio measures the multiplicative effect of a covariate on the baseline hazard; it is interpreted as a relative risk by practitioners, and is frequently reported in clinical and epidemiological studies. The cumulative hazard measures the actual effect of the covariate on the hazard of death, i.e. the importance of mortality due to this covariate. When it is small, the cumulative hazard is interpreted as the difference in outcome incidence due to exposure. In an epidemiological or prevention context, the cumulative hazard is interesting because it accounts both for the importance of the effect of an exposure and for the prevalence of this exposure.

Additive hazards regression models seem to us superior because they allow to directly represent the variation of covariate effects over time, which corresponds to the non-parametric estimation of regression function. By contrast, in the case of multiplicative hazards regression models, the assumption of constant effects must be checked by plotting the Schoenfeld residuals; and when this assumption is not satisfied, an extended Cox model must be performed to model the variation of covariate effects.

As we have seen, our strategy combining multiplicative and additive hazards regression models is interesting because it accounts for different relationships between the hazard function and the covariate. In fact, the two types of models complement each other: by providing different measures of the same effect, they make for a better interpretation of the data. An alternative strategy is the combined multiplicative-additive hazards regression models, as the Cox-Aalen model [24] or the Lin’s additive-multiplicative hazard model [25], in which the covariates are split into two parts according to their - multiplicative or additive - effect on the hazard. However, these models cannot allow to select the type of the effect of the covariates (i.e. additive or multiplicative effect) using the AIC because the likelihood of these models is intractable [25]. We refer the reader to the specific literature on this topic, as this is not the focus of the present article [24–26].

Second, our proposed strategy for optimal fitting of multiplicative and additive hazards regression models is quite easy to implement, and uses diagnostic tools that are available in the major statistical software. As regards multiplicative hazards regression models, our strategy relies not only on classical diagnostic tools (the Schoenfeld and martingale residuals) to check the proportional hazards assumption and the assumption of log-linearity, but also on pseudo-observations and Arjas plots to assess goodness-of-fit. Other approaches for fitting multiplicative hazards regression model have been proposed. Thus, Sasieni and Winnett [16] have introduced a new kind of residuals, the martingale difference residuals, which is used to check both the proportional hazards assumption and the assumption of log-linearity. The limitation of this approach is that it does not require a precise definition of the functional form, even for very large datasets. By contrast, in our strategy, pseudo-observations are used to graphically represent not only the functional form of the continuous covariate but also its variation over time. As such, our strategy helps to select the best functional form for the multivariate model (whether multiplicative or additive). Abrahamowicz and McKenzie [27] have proposed a multiplicative hazards regression model that relaxes both the proportional hazards assumption and the assumption of log-linearity. However, this model is highly flexible, and can consequently result in overfitting. Our approach limits the risk of overfitting by requiring the definition of the functional form and that of the covariate effect – though it should be noted that this can sometimes be time-consuming.

In our strategy, additive hazards regression models are fitted using pseudo-observations, martingale residual processes, and Arjas plots. Other tools have been proposed to assess goodness-of-fit for this type of model. Martinussen and Scheike have introduced two tests based on Gaussian processes to help choose between Aalen’s and Lin’s model in the case of time-invariant effects [28]. Contrary to our approach, in which the assumption of constant effects is checked using a graphical tool, Martinussen and Scheike’s approach has two limitations: the possibility of discordance between the results of the two tests, and the rejection of the null hypothesis when the sample size increases even if the effect is low. McKeague & Utikal [29] have proposed a goodness-of-fit test for Aalen’s model that compares the estimator generated with this model to a non-parametric estimator. The limitation of this approach is that it requires a sample size of at least 1000 subjects to perform satisfactorily. Importantly, goodness-of-fit tests do not give the same information as graphical tools: while the first provide quantitative measures of fit, the second allow for selecting the best functions and functional forms for the multivariate model.

Third, our strategy yields the same results as that proposed by Martinussen and Scheike [21] – who also used the TRACE data frame – although our additive hazards regression model is slightly different from theirs. In our strategy, the multiplicative and additive hazards regression models allow for different interpretations of the same data. Indeed, while the effects of the covariates are significant in both models, they do not act in the same way on the baseline hazard. As regards the covariates sex, clinical heart failure, and ventricular fibrillation, the effect is respectively constant, decreasing, and important then null in both the multiplicative and additive hazards regression models. Here, the variation in mortality due to sex, clinical heart failure, and ventricular fibrillation is given as a relative risk. For the covariate age, both models show a constant effect over time until age 70 and an increasing effect with ageing, but this increasing effect is much greater when given as a cumulative hazard than as a hazard ratio. Thus, the additive hazards regression model better highlights the importance of age as a cause of mortality. As regards the covariate diabetes, the effect is constant in the extended Aalen’s model and increasing in the extended Cox model. In other words, the extended Cox model shows that the relative risk of death increases over time for patients with diabetes, whereas the extended Aalen’s model indicates that mortality due to diabetes is constant over time. The reason for this discrepancy is that diabetic patients initially have higher mortality compared to other patients which results in a sligh decrease of the proportion of diabetic patients relative to the total sample. Thus, to keep the risk of death from diabetes constant, the hazard ratio increases artificially. The above suggests that the additive hazards regression model is a better choice for the analysis of our dataset.

In conclusion, survival analysis is improved by using multiplicative and additive hazards regression models together, but specific steps must be followed to fit the data optimally. Our proposed strategy allows for better interpretation of the data.

## Supplementary Information

**Additional file 1. **R codes used in the proposed strategy for optimal fitting of multiplicative and additive hazards regression models applied to the dataset of patients with myocardial infarction (TRACE data frame in the timereg R package).

## Data Availability

The datasets generated and/or analysed during the current study are available in the timereg R package repository, https://cran.r-project.org/web/packages/timereg/index.html
