# Supplementary material for: A strategy for optimal fitting of multiplicative and additive hazards regression models
Source: BMC Med Res Methodol. 2021 May 6;21:100. doi: 10.1186/s12874-021-01273-2 (PMC8101173; doi:10.1186/s12874-021-01273-2)
Supplement: Supplementary file 1 — Additional file 1. R codes used in the proposed strategy for optimal fitting of multiplicative and additive hazards regression models applied to the dataset of patients with myocardial infarction (TRACE data frame in the timereg R package). [file 12874_2021_1273_MOESM1_ESM.docx]

Additional file 1: R codes used in the proposed strategy for optimal fitting of multiplicative and additive hazards regression models applied to the dataset of patients with myocardial infarction (TRACE data frame in the timereg R package).

library(survival)

library(timereg)

library(ahaz)

data(TRACE)

# Addition of a random number to break the ties

set.seed(906)

TRACE$time=TRACE$time+rnorm(nrow(TRACE),0,0.0001)

# Transformation of the different causes of death into binary covariates (death/censored)

TRACE$status2=as.numeric(TRACE$status!=0)

# Sorting of the time into ascending order

B=TRACE[order(TRACE[,"time"]),]

names(B)=c("id","wmi","status","chf","age","sex","dia","time","vf","status2")

####################################################################################

# Proposed strategy for optimal fitting of multiplicative hazards regression model#

####################################################################################

# Checking the assumption of log-linearity

#######################################

# Martingale residuals

#---------------------

# Null Cox model

mCox0=coxph(Surv(time,status!=0)~1,data=B)

# Martingale residuals with the null Cox model

MartResiduals0=residuals(mCox0,type="martingale")

# Cox model fitted with exp(age/100)

mCoxe=coxph(Surv(time,status!=0)~I(exp(age/100)),data=B)

# Martingale residuals with the Cox model fitted with exp(age/100)

MartResidualse=residuals(mCoxe,type="martingale")

# Figure 1 (plots of martingale residuals)

#~~~~~~~~~~~~~~~~~~~~~~~~~~~~~~~~~~~~~~~~~

x11();par(mfrow=c(1,2))

plot(B$age,MartResiduals0,xlab="age (years)",ylab="Martingale residuals",cex.axis=1.7,cex.lab=1.7,cex.main=2)

abline(h=0,lty=2)

lines(lowess(B$age,MartResiduals0,iter=0))

mtext("a",side=1,adj=0,line=3.5,cex=2.5)

plot(B$age,MartResidualse,xlab="age (years)",ylab="Martingale residuals",cex.axis=1.7,cex.lab=1.7,cex.main=2)

abline(h=0,lty=2)

lines(lowess(B$age,MartResidualse,iter=0))

mtext("b",side=1,adj=0,line=3.5,cex=2.5)

# Cox model fitted with a quadratic effect of age/100

mCox2=coxph(Surv(time,status!=0)~I(age/1000)+I((age/100)^2),data=B)

# Comparisons of AIC of Cox model with exp(age/100) and Cox model with a quadratic effect of age/100

AIC(mCoxe)

AIC(mCox2)

# Checking the proportional hazards assumption

##############################################

# Tests of the correlation between the Schoenfeld residuals and the rank order of event time

mCoxagee=coxph(Surv(time,status!=0)~I(exp(age/100)),data=B)

testagee<-cox.zph(mCoxagee,global=T,transform="rank")

print(testagee)

mCoxsex=coxph(Surv(time,status!=0)~sex,data=B)

testsex<-cox.zph(mCoxsex,global=T,transform="rank")

print(testsex)

mCoxchf=coxph(Surv(time,status!=0)~chf,data=B)

testchf<-cox.zph(mCoxchf,global=T,transform="rank")

print(testchf)

mCoxdia=coxph(Surv(time,status!=0)~dia,data=B)

testdia<-cox.zph(mCoxdia,global=T,transform="rank")

print(testdia)

mCoxvf=coxph(Surv(time,status!=0)~vf,data=B)

testvf<-cox.zph(mCoxvf,global=T,transform="rank")

print(testvf)

# Figure 2 (plots of Schoenfeld residuals)

#~~~~~~~~~~~~~~~~~~~~~~~~~~~~~~~~~~~~~~~~~

x11();par(mfrow=c(2,3))

par(cex.axis=2)

plot(testagee,xlab="",ylab="",main="exp(age/100)",cex.lab=1.7,cex.main=3,lwd=3)

abline(h=mCoxagee$coefficients,lty=3,lwd=2)

mtext("a",side=1,adj=0,line=3.5,cex=2.5)

mtext("Time (years)",side=1,adj=0.5,line=3.5,cex=1.7)

mtext("Beta(t)",side=2,adj=0.5,line=2.2,cex=1.7)

plot(testsex,xlab="",ylab="",main="sex",cex.lab=1.7,cex.main=3,lwd=3)

abline(h=mCoxsex$coefficients,lty=3,lwd=2)

mtext("b",side=1,adj=0,line=3.5,cex=2.5)

mtext("Time (years)",side=1,adj=0.5,line=3.5,cex=1.7)

mtext("Beta(t)",side=2,adj=0.5,line=2.5,cex=1.7)

plot(testchf,xlab="",ylab="",main="chf",cex.lab=1.7,cex.main=3,lwd=3)

abline(h=mCoxchf$coefficients,lty=3,lwd=2)

mtext("c",side=1,adj=0,line=3.5,cex=2.5)

mtext("Time (years)",side=1,adj=0.5,line=3.5,cex=1.7)

mtext("Beta(t)",side=2,adj=0.5,line=2.5,cex=1.7)

plot(testdia,xlab="",ylab="",main="dia",cex.lab=1.7,cex.main=3,lwd=3)

abline(h=mCoxdia$coefficients,lty=3,lwd=2)

mtext("d",side=1,adj=0,line=3.5,cex=2.5)

mtext("Time (years)",side=1,adj=0.5,line=3.5,cex=1.7)

mtext("Beta(t)",side=2,adj=0.5,line=2.2,cex=1.7)

plot(testvf,xlab="",ylab="",main="vf",cex.lab=1.7,cex.main=3,lwd=3)

abline(h=mCoxvf$coefficients,lty=3,lwd=2)

mtext("e",side=1,adj=0,line=3.5,cex=2.5)

mtext("Time (years)",side=1,adj=0.5,line=3.5,cex=1.7)

mtext("Beta(t)",side=2,adj=0.5,line=2.5,cex=1.7)

# Proposed model fitted with a linear time-dependent effect of chf

cut.points=unique(B$time[B$status2==1])

B2=survSplit(data=B,cut=cut.points,end="time",start="time0",event="status2")

B2$chft=B2$chf*B2$time

mCoxchf2=coxph(Surv(time0,time,status2)~chf+chft,data=B2)

summary(mCoxchf2)

# Tests of Schoenfeld residuals

testchf2<-cox.zph(mCoxchf2,global=T,transform="rank")

print(testchf2)

# Proposed model fitted with a break of slope of the effect of vf

# Choice of cut-off

z=NULL

cut.points=unique(B$time[B$status2==1])

B2=survSplit(data=B,cut=cut.points,end="time",start="time0",event="status2")

B2$vft=B2$vf*B2$time

for (i in 1:240){

B2$vft2=B2$vf*(B2$time-i/100)*(B2$time>i/100)

mCoxvf3=coxph(Surv(time0,time,status2)~vf+vft+vft2,data=B2)

z[i]=AIC(mCoxvf3)

cat(i,"\n");flush.console()

}

# Cut-off

which(z==min(z))/100

# Extended Cox model fitted with the best cut-off

cut.points=unique(B$time[B$status2==1])

B2=survSplit(data=B,cut=cut.points,end="time",start="time0",event="status2")

B2$vft=B2$vf*B2$time

B2$vft2=B2$vf*(B2$time-0.15)*(B2$time>0.15)

mCoxvf3=coxph(Surv(time0,time,status2)~vf+vft+vft2,data=B2)

summary(mCoxvf3)

testmCoxvf3=cox.zph(mCoxvf3,global=T,transform="km")

print(testmCoxvf3)

# Assessing goodness-of-fit

##############################

# Compute pseudo-observations

#----------------------------

B$censoring=as.numeric(B$status!=0)

KM0=matrix(0,nrow=nrow(B),ncol=nrow(B)-1)

for (i in 1:nrow(B)){B2=B[-i,]

for(j in 1:nrow(B2)){

KM0[i,j]=1-B2$censoring[j]/(nrow(B2)-j+1)}}

S0=matrix(0,nrow=nrow(B),ncol=nrow(B)-1)

for (i in 1:nrow(B)){S0[i,]=cumprod(KM0[i,])}

S=matrix(0,nrow=nrow(B),ncol=nrow(B))

S[1,2:nrow(B)]=S0[1,]

S[nrow(B),1:(nrow(B)-1)]=S0[nrow(B),]

S[nrow(B),nrow(B)]=S0[nrow(B),nrow(B)-1]

for (i in 2:(nrow(B)-1)){

S[i,1:(i-1)]=S0[i,1:(i-1)]

S[i,i]=S0[i,i-1]

S[i,(i+1):(nrow(B))]=S0[i,i:(nrow(B)-1)]}

S[1,1]=1

KMt=rep(0,nrow(B))

for (i in 1:nrow(B)){KMt[i]=1-B$censoring[i]/(nrow(B)-i+1)}

St=rep(0,nrow(B))

St=cumprod(KMt)

matS=matrix(rep(nrow(B)*St,nrow(B)),nrow=nrow(B),ncol=nrow(B),byrow=T)

POS=matS-(nrow(B)-1)*S

# Estimation of the nine deciles of the event time distribution

times<-unique(B$time[B$status!=0])

tps=round(c(length(times)/10,2*length(times)/10,3*length(times)/10,4*length(times)/10,5*length(times)/10,6*length(times)/10,7*length(times)/10,8*length(times)/10,9*length(times)/10),0)

# Figure 3 (plot of pseudo-observations)

#~~~~~~~~~~~~~~~~~~~~~~~~~~~~~~~~~~~~~~~

x11()

plot(log(-log(lowess(B$age/100,POS[,tps[1]],iter=0)$y))~lowess(B$age/100,POS[,tps[1]],iter=0)$x,xlab="age/100 (years)",ylab="",type="l",ylim=c(-7.2,1.2),cex.axis=1.7,lwd=3,cex.lab=1.7,col="grey0")

points(0.682305,-2.998537,pch=0,lwd=3,cex=1.5,col="grey0")

lines(log(-log(lowess(B$age/100,POS[,tps[2]],iter=0)$y))~lowess(B$age/100,POS[,tps[2]],iter=0)$x,type="l",col="grey9",lwd=3)

points(0.682305,-2.337397,pch=1,lwd=3,cex=1.5,col="grey9")

lines(log(-log(lowess(B$age/100,POS[,tps[3]],iter=0)$y))~lowess(B$age/100,POS[,tps[3]],iter=0)$x,type="l",col="grey18",lwd=3)

points(0.682305,-1.901599,pch=2,lwd=3,cex=1.5,col="grey18")

lines(log(-log(lowess(B$age/100,POS[,tps[4]],iter=0)$y))~lowess(B$age/100,POS[,tps[4]],iter=0)$x,type="l",col="grey27",lwd=3)

points(0.682305,-1.586416,pch=3,lwd=3,cex=1.5,col="grey27")

lines(log(-log(lowess(B$age/100,POS[,tps[5]],iter=0)$y))~lowess(B$age/100,POS[,tps[5]],iter=0)$x,type="l",col="grey36",lwd=3)

points(0.682305,-1.28576,pch=4,lwd=3,cex=1.5,col="grey36")

lines(log(-log(lowess(B$age/100,POS[,tps[6]],iter=0)$y))~lowess(B$age/100,POS[,tps[6]],iter=0)$x,type="l",col="grey45",lwd=3)

points(0.682305,-1.046544,pch=5,lwd=3,cex=1.5,col="grey45")

lines(log(-log(lowess(B$age/100,POS[,tps[7]],iter=0)$y))~lowess(B$age/100,POS[,tps[7]],iter=0)$x,type="l",col="grey54",lwd=3)

points(0.682305,-0.8717967,pch=6,lwd=3,cex=1.5,col="grey54")

lines(log(-log(lowess(B$age/100,POS[,tps[8]],iter=0)$y))~lowess(B$age/100,POS[,tps[8]],iter=0)$x,type="l",col="grey63",lwd=3)

points(0.682305,-0.6818521,pch=7,lwd=3,cex=1.5,col="grey63")

lines(log(-log(lowess(B$age/100,POS[,tps[9]],iter=0)$y))~lowess(B$age/100,POS[,tps[9]],iter=0)$x,type="l",col="grey72",lwd=3)

points(0.682305,-0.4755059,pch=8,lwd=3,cex=1.5,col="grey72")

abline(h=0)

legend("bottomright",c("1st decile (0.03 year)","2nd decile (0.09 year)","3rd decile (0.37 year)","4th decile (0.97 year)","5th decile (1.73 year)","6th decile (2.46 years)","7th decile (3.46 years)","8th decile (4.61 years)","9th decile (5.81 years)"),col=c("grey0","grey9","grey18","grey27","grey36","grey45","grey54","grey63","grey72"),lwd=3,cex=1,pch=0:8,pt.cex=1.5)

mtext("cloglog(Pseudo-observations)",side=2,adj=0.5,line=2.5,cex=1.7)

# Arjas plots

#-------------

# Cox model fitted with age

mCoxage=coxph(Surv(time,status!=0)~age,data=B)

Lambdaage=matrix(0,nrow=nrow(B),ncol=nrow(B))

for (i in 1:nrow(B)){

Lambdaage[i,1:i]=survfit(mCoxage,newdata=data.frame(age=B$age[i]))$cumhaz[1:i]

cat(i,"\n");flush.console()}

for(i in 1:(nrow(B)-1)){Lambdaage[i,(i+1):nrow(B)]=Lambdaage[i,i]}

# Cox model fitted with exp(age/100)

mCoxagee=coxph(Surv(time,status!=0)~I(exp(age/100)),data=B)

Lambdaagee=matrix(0,nrow=nrow(B),ncol=nrow(B))

for (i in 1:nrow(B)){

Lambdaagee[i,1:i]=survfit(mCoxagee,newdata=data.frame(age=B$age[i]))$cumhaz[1:i]

cat(i,"\n");flush.console()}

for(i in 1:(nrow(B)-1)){Lambdaagee[i,(i+1):nrow(B)]=Lambdaagee[i,i]}

# Figure 4 (Arjas plots for age)

#~~~~~~~~~~~~~~~~~~~~~~~~~~~~~~~~

x11();par(mfrow=c(1,2))

B$agecat=cut(B$age,breaks=c(20,59.61,68.23,75.39,100))

plot(colSums(Lambdaage[which(B$agecat=="(20,59.6]"),])[B$agecat=="(20,59.6]"&B$status2==1]~cumsum(summary(survfit(Surv(time,status2)~1,data=B,subset=(agecat=="(20,59.6]")))$n.event),type="l",xlim=c(0,400),ylim=c(0,400),lty=2,lwd=5,col="grey0",xlab="Number of observed events",ylab="Number of estimated events",cex.lab=1.7,,cex.axis=1.7)

lines(colSums(Lambdaage[which(B$agecat=="(59.6,68.2]"),])[B$agecat=="(59.6,68.2]"&B$status2==1]~cumsum(summary(survfit(Surv(time,status2)~1,data=B,subset=(agecat=="(59.6,68.2]")))$n.event),type="l",lty=2,lwd=5,col="grey30")

lines(colSums(Lambdaage[which(B$agecat=="(68.2,75.4]"),])[B$agecat=="(68.2,75.4]"&B$status2==1]~cumsum(summary(survfit(Surv(time,status2)~1,data=B,subset=(agecat=="(68.2,75.4]")))$n.event),type="l",lty=2,lwd=5,col="grey60")

lines(colSums(Lambdaage[which(B$agecat=="(75.4,100]"),])[B$agecat=="(75.4,100]"&B$status2==1]~cumsum(summary(survfit(Surv(time,status2)~1,data=B,subset=(agecat=="(75.4,100]")))$n.event),type="l",lty=2,lwd=5,col="grey90")

abline(0,1)

legend(0,400,c(expression("age"<="59.6 years"),"age=(59.6,68.2] years","age=(68.2,75.4] years","age>75.4 years"),lty=2,lwd=5,col=c("grey0","grey30","grey60","grey90"),cex=1.7)

mtext("a",side=1,adj=0,line=3.5,cex=2.5)

plot(colSums(Lambdaagee[which(B$agecat=="(20,59.6]"),])[B$agecat=="(20,59.6]"&B$status2==1]~cumsum(summary(survfit(Surv(time,status2)~1,data=B,subset=(agecat=="(20,59.6]")))$n.event),type="l",xlim=c(0,400),ylim=c(0,400),lty=2,lwd=5,col="grey0",xlab="Number of observed events",ylab="Number of estimated events",cex.lab=1.7,cex.axis=1.7)

lines(colSums(Lambdaagee[which(B$agecat=="(59.6,68.2]"),])[B$agecat=="(59.6,68.2]"&B$status2==1]~cumsum(summary(survfit(Surv(time,status2)~1,data=B,subset=(agecat=="(59.6,68.2]")))$n.event),type="l",lty=2,lwd=5,col="grey30")

lines(colSums(Lambdaagee[which(B$agecat=="(68.2,75.4]"),])[B$agecat=="(68.2,75.4]"&B$status2==1]~cumsum(summary(survfit(Surv(time,status2)~1,data=B,subset=(agecat=="(68.2,75.4]")))$n.event),type="l",lty=2,lwd=5,col="grey60")

lines(colSums(Lambdaagee[which(B$agecat=="(75.4,100]"),])[B$agecat=="(75.4,100]"&B$status2==1]~cumsum(summary(survfit(Surv(time,status2)~1,data=B,subset=(agecat=="(75.4,100]")))$n.event),type="l",lty=2,lwd=5,col="grey90")

abline(0,1)

legend(0,400,c(expression("age"<="59.6 years"),"age=(59.6,68.2] years","age=(68.2,75.4] years","age>75.4 years"),lty=2,lwd=5,col=c("grey0","grey30","grey60","grey90"),cex=1.7)

mtext("b",side=1,adj=0,line=3.5,cex=2.5)

# Cox model fitted with chf

mCoxchf=coxph(Surv(time,status!=0)~chf,data=B)

Lambdachf=matrix(0,nrow=nrow(B),ncol=nrow(B))

for (i in 1:nrow(B)){

Lambdachf[i,1:i]=survfit(mCoxchf,newdata=data.frame(chf=B$chf[i]))$cumhaz[1:i]

cat(i,"\n");flush.console()}

for(i in 1:(nrow(B)-1)){Lambdachf[i,(i+1):nrow(B)]=Lambdachf[i,i]}

# Cox model fitted with chf with time-dependent effect

cut.points=unique(B$time[B$status2==1])

B2=survSplit(data=B,cut=cut.points,end="time",start="time0",event="status2")

B2$chft=B2$chf*B2$time

mCoxchf2=coxph(Surv(time0,time,status2)~chf+chft,data=B2)

summary(mCoxchf2)

der=B2$id[which.max(B2$time)]

intervals=B2[B2$id==der,c("time0","time","status2")]

covs=data.frame(chf=1,intervals)

covs$chft=covs$chf*covs$time

cumhaz=matrix(0,nrow=2,ncol=nrow(B))

cumhaz[1,1:length(survfit(mCoxchf2,newdata=data.frame(chf=0,chft=0))$cumhaz)]=survfit(mCoxchf2,newdata=data.frame(chf=0,chft=0))$cumhaz

cumhaz[2,1:length(survfit(mCoxchf2,newdata=covs,individual=TRUE)$cumhaz)]=survfit(mCoxchf2,newdata=covs,individual=TRUE)$cumhaz

for (i in 2:nrow(B)){if(cumhaz[1,i]==0){cumhaz[1,i]=cumhaz[1,i-1]}}

for (i in 2:nrow(B)){if(cumhaz[2,i]==0){cumhaz[2,i]=cumhaz[2,i-1]}}

Lambdachfdt=matrix(0,nrow=nrow(B),ncol=nrow(B))

for (i in 1:nrow(B)){

if(B$chf[i]==0)

Lambdachfdt[i,1:i]=cumhaz[1,1:i]

else{Lambdachfdt[i,1:i]=cumhaz[2,1:i]}

cat(i,"\n");flush.console()}

for(i in 1:(nrow(B)-1)){Lambdachfdt[i,(i+1):nrow(B)]=Lambdachfdt[i,i]}

# Figure 5 (Arjas plots for chf)

#~~~~~~~~~~~~~~~~~~~~~~~~~~~~~~~~

x11();par(mfrow=c(1,2))

plot(colSums(Lambdachf[which(B$chf==1),])[B$chf==1&B$status2==1]~cumsum(summary(survfit(Surv(time,status2)~1,data=B,subset=(chf==1)))$n.event),type="l",xlim=c(0,700),ylim=c(0,700),lty=2,lwd=5,col="grey0",xlab="Number of observed events",ylab="Number of estimated events",cex.lab=1.7,cex.axis=1.7)

lines(colSums(Lambdachf[which(B$chf==0),])[B$chf==0&B$status2==1]~cumsum(summary(survfit(Surv(time,status2)~1,data=B,subset=(chf==0)))$n.event),type="l",lty=2,lwd=5,col="grey70")

abline(0,1)

legend(0,700,c("Presence of chf","Absence of chf"),lty=2,lwd=5,col=c("grey0","grey70"),cex=1.7)

mtext("a",side=1,adj=0,line=3.5,cex=2.5)

plot(colSums(Lambdachfdt[which(B$chf==1),])[B$chf==1&B$status2==1]~cumsum(summary(survfit(Surv(time,status2)~1,data=B,subset=(chf==1)))$n.event),type="l",xlim=c(0,700),ylim=c(0,700),lty=2,lwd=5,col="grey0",xlab="Number of observed events",ylab="Number of estimated events",cex.lab=1.7,cex.axis=1.7)

lines(colSums(Lambdachfdt[which(B$chf==0),])[B$chf==0&B$status2==1]~cumsum(summary(survfit(Surv(time,status2)~1,data=B,subset=(chf==0)))$n.event),type="l",lty=2,lwd=5,col="grey70")

abline(0,1)

legend(0,700,c("Presence of chf","Absence of chf"),lty=2,lwd=5,col=c("grey0","grey70"),cex=1.7)

mtext("b",side=1,adj=0,line=3.5,cex=2.5)

# Multivariate Cox model

#-----------------------

cut.points=unique(B$time[B$status2==1])

B2=survSplit(data=B,cut=cut.points,end="time",start="time0",event="status2")

B2$chft=B2$chf*B2$time

B2$vft=B2$vf*B2$time

B2$vft2=B2$vf*(B2$time-0.15)*(B2$time>0.15)

mCoxm=coxph(Surv(time0,time,status2)~I(exp(age/100))+sex+chf+chft+dia+vf+vft+vft2,data=B2)

summary(mCoxm)

testCoxm=cox.zph(mCoxm,transform="km")

testCoxm

x11();par(mfrow=c(2,4));plot(testCoxm)

x11();plot(testCoxm[5])

B2$diat=B2$dia*B2$time

mCoxm2=coxph(Surv(time0,time,status2)~I(exp(age/100))+sex+chf+chft+dia+diat+vf+vft+vft2,data=B2)

summary(mCoxm2)

testCoxm2=cox.zph(mCoxm2,transform="km")

testCoxm2

# Figure 6 (hazard ratio for age)

#~~~~~~~~~~~~~~~~~~~~~~~~~~~~~~~~~

x11()

j=function(x){exp(mCoxm2$coefficient[1]*exp(x/100))/exp(mCoxm2$coefficient[1]*exp((x-1)/100))}

jinf=function(x){exp((mCoxm2$coefficient[1]-1.96*sqrt(mCoxm2$var[1,1]))*exp(x/100))/exp((mCoxm2$coefficient[1]-1.96*sqrt(mCoxm2$var[1,1]))*exp((x-1)/100))}

jsup=function(x){exp((mCoxm2$coefficient[1]+1.96*sqrt(mCoxm2$var[1,1]))*exp(x/100))/exp((mCoxm2$coefficient[1]+1.96*sqrt(mCoxm2$var[1,1]))*exp((x-1)/100))}

curve(j,24,97,ylim=c(0.95,1.1),cex.axis=1.7,lwd=3,cex.lab=1.7,xlab="age (years)",ylab="Hazard ratio",cex.main=2,main="")

curve(jinf,add=T,lty=2,lwd=3)

curve(jsup,add=T,lty=2,lwd=3)

abline(h=1,lty=3)

# Figure 7 (hazard ratios for chf, dia and vf)

#~~~~~~~~~~~~~~~~~~~~~~~~~~~~~~~~~~~~~~~~~~~~~~

x11();

par(mfrow=c(1,3))

# Hazard ratio for chf

f=function(x){exp(mCoxm2$coefficient[3]+mCoxm2$coefficient[4]*x)}

finf=function(x){exp(mCoxm2$coefficient[3]+mCoxm2$coefficient[4]*x-1.96*sqrt(mCoxm2$var[3,3]+x^2*mCoxm2$var[4,4]+2*x*mCoxm2$var[3,4]))}

fsup=function(x){exp(mCoxm2$coefficient[3]+mCoxm2$coefficient[4]*x+1.96*sqrt(mCoxm2$var[3,3]+x^2*mCoxm2$var[4,4]+2*x*mCoxm2$var[3,4]))}

curve(f,0,8.5,ylim=c(0,4),cex.axis=1.7,lwd=3,cex.lab=2,cex.axis=2,xlab="Time (years)",ylab="",cex.main=3,main="chf")

curve(finf,add=T,lty=2,lwd=3)

curve(fsup,add=T,lty=2,lwd=3)

abline(h=1,lty=3)

mtext("Hazard ratio",side=2,adj=0.5,line=2.3,cex=1.7)

mtext("a",side=1,adj=0,line=3.5,cex=1.8)

# Hazard ratio for dia

g=function(x){exp(mCoxm2$coefficient[5]+mCoxm2$coefficient[6]*x)}

ginf=function(x){exp(mCoxm2$coefficient[5]+mCoxm2$coefficient[6]*x-1.96*sqrt(mCoxm2$var[5,5]+x^2*mCoxm2$var[6,6]+2*x*mCoxm2$var[5,6]))}

gsup=function(x){exp(mCoxm2$coefficient[5]+mCoxm2$coefficient[6]*x+1.96*sqrt(mCoxm2$var[5,5]+x^2*mCoxm2$var[6,6]+2*x*mCoxm2$var[5,6]))}

curve(g,0,8.5,ylim=c(0,6),cex.axis=1.7,lwd=3,cex.lab=2,cex.axis=2,xlab="Time (years)",ylab="",cex.main=3,main="dia")

curve(ginf,add=T,lty=2,lwd=3)

curve(gsup,add=T,lty=2,lwd=3)

abline(h=1,lty=3)

mtext("Hazard ratio",side=2,adj=0.5,line=2.3,cex=1.7)

mtext("b",side=1,adj=0,line=3.5,cex=1.8)

# Hazard ratio for vf

h=function(x){exp(mCoxm2$coefficient[7]+x*mCoxm2$coefficient[8]+(x-0.15)*mCoxm2$coefficient[9]*(x>0.15))}

hinf=function(x){exp(mCoxm2$coefficient[7]+x*mCoxm2$coefficient[8]+(x-0.15)*mCoxm2$coefficient[9]*(x>0.15)-1.96*sqrt(mCoxm2$var[7,7]+x^2*mCoxm2$var[8,8]+2*x*mCoxm2$var[7,8]+(x>0.15)*((x-0.15)^2*mCoxm2$var[9,9]+2*(x-0.15)*mCoxm2$var[7,9]+2*x*(x-0.15)*mCoxm2$var[8,9])))}

hsup=function(x){exp(mCoxm2$coefficient[7]+x*mCoxm2$coefficient[8]+(x-0.15)*mCoxm2$coefficient[9]*(x>0.15)+1.96*sqrt(mCoxm2$var[7,7]+x^2*mCoxm2$var[8,8]+2*x*mCoxm2$var[7,8]+(x>0.15)*((x-0.15)^2*mCoxm2$var[9,9]+2*(x-0.15)*mCoxm2$var[7,9]+2*x*(x-0.15)*mCoxm2$var[8,9])))}

curve(h,0,8.5,ylim=c(0,14),cex.axis=1.7,lwd=3,cex.lab=2,cex.axis=2,xlab="Time (years)",ylab="",cex.main=3,main="vf")

curve(hinf,add=T,lty=2,lwd=3)

curve(hsup,add=T,lty=2,lwd=3)

abline(h=1,lty=3)

mtext("Hazard ratio",side=2,adj=0.5,line=2.3,cex=1.7)

mtext("c",side=1,adj=0,line=3.5,cex=1.8)

###############################################################################

# Proposed strategy for optimal fitting of additive hazards regression models #

###############################################################################

# Checking the assumption of linearity

################################

# Pseudo-observations

#--------------------

# Figure 8 (plot of pseudo-observations)

#~~~~~~~~~~~~~~~~~~~~~~~~~~~~~~~~~~~~~~~

x11()

plot(-log(lowess(B$age,POS[,tps[1]],iter=0)$y)/times[tps[1]]~lowess(B$age,POS[,tps[1]],iter=0)$x,xlab="age (years)",ylab="",type="l",ylim=c(-1,7),cex.axis=1.7,lwd=3,cex.lab=1.7,col="grey0")

points(68.2305,1.994219,pch=0,lwd=3,cex=1.5,col="grey0")

lines(-log(lowess(B$age,POS[,tps[2]],iter=0)$y)/times[tps[2]]~lowess(B$age,POS[,tps[2]],iter=0)$x,type="l",col="grey10",lwd=3)

points(68.2305,1.061049,pch=1,lwd=3,cex=1.5,col="grey10")

lines(-log(lowess(B$age,POS[,tps[3]],iter=0)$y)/times[tps[3]]~lowess(B$age,POS[,tps[3]],iter=0)$x,type="l",col="grey20",lwd=3)

points(68.2305,0.4040244,pch=2,lwd=3,cex=1.5,col="grey20")

lines(-log(lowess(B$age,POS[,tps[4]],iter=0)$y)/times[tps[4]]~lowess(B$age,POS[,tps[4]],iter=0)$x,type="l",col="grey30",lwd=3)

points(68.2305,0.2114368,pch=3,lwd=3,cex=1.5,col="grey30")

lines(-log(lowess(B$age,POS[,tps[5]],iter=0)$y)/times[tps[5]]~lowess(B$age,POS[,tps[5]],iter=0)$x,type="l",col="grey40",lwd=3)

points(68.2305,0.1600768,pch=4,lwd=3,cex=1.5,col="grey40")

lines(-log(lowess(B$age,POS[,tps[6]],iter=0)$y)/times[tps[6]]~lowess(B$age,POS[,tps[6]],iter=0)$x,type="l",col="grey50",lwd=3)

points(68.2305,0.1425707,pch=5,lwd=3,cex=1.5,col="grey50")

lines(-log(lowess(B$age,POS[,tps[7]],iter=0)$y)/times[tps[7]]~lowess(B$age,POS[,tps[7]],iter=0)$x,type="l",col="grey60",lwd=3)

points(68.2305,0.1209347,pch=6,lwd=3,cex=1.5,col="grey60")

lines(-log(lowess(B$age,POS[,tps[8]],iter=0)$y)/times[tps[8]]~lowess(B$age,POS[,tps[8]],iter=0)$x,type="l",col="grey70",lwd=3)

points(68.2305,0.1097193,pch=7,lwd=3,cex=1.5,col="grey70")

lines(-log(lowess(B$age,POS[,tps[9]],iter=0)$y)/times[tps[9]]~lowess(B$age,POS[,tps[9]],iter=0)$x,type="l",col="grey80",lwd=3)

points(68.2305,0.1069086,pch=8,lwd=3,cex=1.5,col="grey80")

abline(h=0)

legend("topleft",c("1st decile (0.03 year)","2nd decile (0.09 year)","3rd decile (0.37 year)","4th decile (0.97 year)","5th decile (1.73 year)","6th decile (2.46 years)","7th decile (3.46 years)","8th decile (4.61 years)","9th decile (5.81 years)"),col=c("grey0","grey9","grey18","grey27","grey36","grey45","grey54","grey63","grey72"),lwd=3,cex=1,pch=0:8,pt.cex=1.5)

mtext("-log(Pseudo-observations)/t",side=2,adj=0.5,line=2.5,cex=1.7)

# Martingale residual processes

#------------------------------

B$age2=cut(B$age,c(22.8,59.6,68.2,75.4,96.4),include.lowest=T)

MRP=function(event,covariates,contcat){

I=diag(as.numeric(event))

Z=rep(0,nrow(covariates)*(ncol(covariates)+1)*nrow(covariates))

dim(Z)=c(nrow(covariates),(ncol(covariates)+1),nrow(covariates))

Z[,1,]=1

for(i in 2:(ncol(covariates)+1)){

Z[,i,]=covariates[,i-1]}

for (i in 2:nrow(covariates)){Z[1:i-1,,i]=0}

beta=matrix(0,nrow=dim(Z)[2],ncol=nrow(covariates))

for (i in 1:nrow(covariates)){

beta[,i]=tryCatch(solve(crossprod(Z[,,i],Z[,,i]))%*%t(Z[,,i])%*%I[i,],warning=function(e) F,error=function(e) rep(0,ncol(Z)))}

Beta=matrix(0,nrow=dim(Z)[2],ncol=nrow(covariates))

for(i in 1:dim(Z)[2]){Beta[i,]=cumsum(beta[i,])}

M=matrix(0,nrow=nrow(covariates),ncol=nrow(covariates))

J=diag(nrow(covariates))

for (i in 1:nrow(covariates)){

M[,i]=tryCatch((J-Z[,,i]%*%solve(crossprod(Z[,,i],Z[,,i]))%*%t(Z[,,i]))%*%I[i,],warning=function(e) F,error=function(e) rep(0,nrow(covariates)))}

M2=matrix(0,nrow=nrow(covariates),ncol=nrow(covariates))

for(i in 1:nrow(covariates)){M2[i,]=cumsum(M[i,])}

model.matrix(~as.factor(contcat)-1)->contcatq

Mt<<-t(as.matrix(contcatq))%*%M2

V=rep(0,nrow(covariates)*nlevels(contcat)*nlevels(contcat))

dim(V)=c(nlevels(contcat),nlevels(contcat),nrow(covariates))

for (i in 1:nrow(covariates)){

V[,,i]=tryCatch(t(as.matrix(contcatq))%*%(J-Z[,,i]%*%solve(crossprod(Z[,,i],Z[,,i]))%*%t(Z[,,i]))%*%diag(I[i,])%*%t(J-Z[,,i]%*%solve(crossprod(Z[,,i],Z[,,i]))%*%t(Z[,,i]))%*%as.matrix(contcatq),warning=function(e) F,error=function(e) matrix(0,nlevels(contcat),nlevels(contcat)))}

V2=rep(0,nrow(covariates)*nlevels(contcat)*nlevels(contcat))

dim(V2)=c(nlevels(contcat),nlevels(contcat),nrow(covariates))

for(i in 1:nlevels(contcat)){for (j in 1:nlevels(contcat)){V2[i,j,]<-cumsum(V[i,j,])}}

V2<<-V2

for (i in 1:nlevels(contcat)){

print(1-pchisq(t(Mt[i,nrow(covariates)])%*%solve(V2[i,i,nrow(covariates)])%*%Mt[i,nrow(covariates)],1))}

print(1-pchisq(t(Mt[1:(nlevels(contcat)-1),nrow(covariates)])%*%solve(V2[1:(nlevels(contcat)-1),1:(nlevels(contcat)-1),nrow(covariates)])%*%Mt[1:(nlevels(contcat)-1),nrow(covariates)],(nlevels(contcat)-1)))

}

MRP(event=B$status2,covariates=matrix(exp(B$age/10),nrow(B),ncol=1),contcat=B$age2)

# Figure 9 (plots of martingale residual processes)

#~~~~~~~~~~~~~~~~~~~~~~~~~~~~~~~~~~~~~~~~~~~~~~~~~~~

x11();par(mfrow=c(1,2))

plot(Mt[1,]~B$time,type="l",ylim=c(-80,90),xlab="Time (years)",ylab="Martingale residual processes",col="grey0",cex.axis=1.5,cex.lab=1.5,lwd=3)

lines(Mt[2,]~B$time,type="l",lty=1,col="grey30",lwd=3)

lines(Mt[3,]~B$time,type="l",lty=1,col="grey60",lwd=3)

lines(Mt[4,]~B$time,type="l",lty=1,col="grey90",lwd=3)

legend(0,90,c(expression("age"<="59.6 years"),"age=(59.6,68.2] years","age=(68.2,75.4] years","age>75.4 years"),lty=1,lwd=5,col=c("grey0","grey30","grey60","grey90"),cex=1.7)

mtext("a",side=1,adj=0,line=3.5,cex=2.5)

lines((Mt[1,]-1.96*sqrt(V2[1,1,]))~B$time,type="l",lty=2,col="grey0",lwd=3)

lines((Mt[1,]+1.96*sqrt(V2[1,1,]))~B$time,type="l",lty=2,col="grey0",lwd=3)

lines((Mt[2,]-1.96*sqrt(V2[2,2,]))~B$time,type="l",lty=2,col="grey30",lwd=3)

lines((Mt[2,]+1.96*sqrt(V2[2,2,]))~B$time,type="l",lty=2,col="grey30",lwd=3)

lines((Mt[3,]-1.96*sqrt(V2[3,3,]))~B$time,type="l",lty=2,col="grey60",lwd=3)

lines((Mt[3,]+1.96*sqrt(V2[3,3,]))~B$time,type="l",lty=2,col="grey60",lwd=3)

lines((Mt[4,]-1.96*sqrt(V2[4,4,]))~B$time,type="l",lty=2,col="grey90",lwd=3)

lines((Mt[4,]+1.96*sqrt(V2[4,4,]))~B$time,type="l",lty=2,col="grey90",lwd=3)

abline(h=0)

MRP(event=B$status2,covariates=matrix(c(exp(B$age/10),exp(B$age/10-7)*(B$age>70)),nrow(B),ncol=2),contcat=B$age2)

plot(Mt[1,]~B$time,type="l",ylim=c(-80,90),xlab="Time (years)",ylab="Martingale residual processes",col="grey0",cex.axis=1.5,cex.lab=1.5,lwd=3)

lines(Mt[2,]~B$time,type="l",lty=1,col="grey30",lwd=3)

lines(Mt[3,]~B$time,type="l",lty=1,col="grey60",lwd=3)

lines(Mt[4,]~B$time,type="l",lty=1,col="grey90",lwd=3)

legend(0,90,c(expression("age"<="59.6 years"),"age=(59.6,68.2] years","age=(68.2,75.4] years","age>75.4 years"),lty=1,lwd=5,col=c("grey0","grey30","grey60","grey90"),cex=1.7)

mtext("b",side=1,adj=0,line=3.5,cex=2.5)

lines((Mt[1,]-1.96*sqrt(V2[1,1,]))~B$time,type="l",lty=2,col="grey0",lwd=3)

lines((Mt[1,]+1.96*sqrt(V2[1,1,]))~B$time,type="l",lty=2,col="grey0",lwd=3)

lines((Mt[2,]-1.96*sqrt(V2[2,2,]))~B$time,type="l",lty=2,col="grey30",lwd=3)

lines((Mt[2,]+1.96*sqrt(V2[2,2,]))~B$time,type="l",lty=2,col="grey30",lwd=3)

lines((Mt[3,]-1.96*sqrt(V2[3,3,]))~B$time,type="l",lty=2,col="grey60",lwd=3)

lines((Mt[3,]+1.96*sqrt(V2[3,3,]))~B$time,type="l",lty=2,col="grey60",lwd=3)

lines((Mt[4,]-1.96*sqrt(V2[4,4,]))~B$time,type="l",lty=2,col="grey90",lwd=3)

lines((Mt[4,]+1.96*sqrt(V2[4,4,]))~B$time,type="l",lty=2,col="grey90",lwd=3)

abline(h=0)

# Checking the assumption of constant effects

#########################################

# Aalen's and Lin's models

# age

Amage=aareg(Surv(time,status!=0)~I(exp(age/10))+I(exp((age/10-7))*(age>70)),data=B)

Linmage<-ahaz(Surv(B$time,B$status!=0),as.matrix(cbind(exp(B$age/10),exp(B$age/10-7)*(B$age>70))))

# sex

Amsex=aareg(Surv(time,status!=0)~sex,data=B)

Linmsex<-ahaz(Surv(B$time,B$status!=0),as.matrix(B$sex))

# chf

Amchf=aareg(Surv(time,status!=0)~chf,data=B)

Linmchf<-ahaz(Surv(B$time,B$status!=0),as.matrix(B$chf))

# dia

Amdia=aareg(Surv(time,status!=0)~dia,data=B)

Linmdia<-ahaz(Surv(B$time,B$status!=0),as.matrix(B$dia))

# vf

Amvf=aareg(Surv(time,status!=0)~vf,data=B)

Linmvf<-ahaz(Surv(B$time,B$status!=0),as.matrix(B$vf))

# Figure 10 (plots of cumulative hazards)

#~~~~~~~~~~~~~~~~~~~~~~~~~~~~~~~~~~~~~~~~

x11();par(mfrow=c(2,3))

plot(Amage[2],cex.axis=2,cex.main=3,main="exp(age/10)",col.lab="white")

abline(0,summary(Linmage)$coefficients[1,1],col=1,lty=2)

mtext("a",side=1,adj=0,line=3.5,cex=2.5)

mtext("Time (years)",side=1,adj=0.5,line=3.5,cex=1.7)

mtext("Beta(t)",side=2,adj=0.5,line=2.2,cex=1.7)

plot(Amage[3],cex.axis=2,cex.main=3,main="exp(age/10-7)*(age>70)",col.lab="white")

abline(0,summary(Linmage)$coefficients[2,1],col=1,lty=2)

mtext("b",side=1,adj=0,line=3.5,cex=2.5)

mtext("Time (years)",side=1,adj=0.5,line=3.5,cex=1.7)

mtext("Beta(t)",side=2,adj=0.5,line=2.5,cex=1.7)

plot(Amsex[2],cex.axis=2,cex.main=3,main="sex",col.lab="white")

abline(0,summary(Linmsex)$coefficients[1,1],col=1,lty=2)

mtext("c",side=1,adj=0,line=3.5,cex=2.5)

mtext("Time (years)",side=1,adj=0.5,line=3.5,cex=1.7)

mtext("Beta(t)",side=2,adj=0.5,line=2.5,cex=1.7)

plot(Amchf[2],cex.axis=2,cex.main=3,main="chf",col.lab="white",ylim=c(0,0.8))

abline(0,summary(Linmchf)$coefficients[1,1],col=1,lty=2)

mtext("d",side=1,adj=0,line=3.5,cex=2.5)

mtext("Time (years)",side=1,adj=0.5,line=3.5,cex=1.7)

mtext("Beta(t)",side=2,adj=0.5,line=2.2,cex=1.7)

plot(Amdia[2],cex.axis=2,cex.main=3,main="dia",col.lab="white",ylim=c(0,0.8))

abline(0,summary(Linmdia)$coefficients[1,1],col=1,lty=2)

mtext("e",side=1,adj=0,line=3.5,cex=2.5)

mtext("Time (years)",side=1,adj=0.5,line=3.5,cex=1.7)

mtext("Beta(t)",side=2,adj=0.5,line=2.5,cex=1.7)

plot(Amvf[2],cex.axis=2,cex.main=3,main="vf",col.lab="white",ylim=c(0,0.8))

abline(0,summary(Linmvf)$coefficients[1,1],col=1,lty=2)

mtext("f",side=1,adj=0,line=3.5,cex=2.5)

mtext("Time (years)",side=1,adj=0.5,line=3.5,cex=1.7)

mtext("Beta(t)",side=2,adj=0.5,line=2.5,cex=1.7)

# Assessing goodness-of-fit

##############################

# Arjas plots

#-------------

# Figure 11 (Arjas plots)

#~~~~~~~~~~~~~~~~~~~~~~~~~

I=diag(as.numeric(B$status!=0))

Z=rep(0,nrow(B)*2*nrow(B))

dim(Z)=c(nrow(B),2,nrow(B))

Z[,1,]=1

Z[,2,]=B$age

for (i in 2:nrow(B)){

Z[1:i-1,,i]=0}

beta=matrix(0,nrow=dim(Z)[2],ncol=nrow(B))

for (i in 1:nrow(B)){

beta[,i]=tryCatch(solve(crossprod(Z[,,i],Z[,,i]))%*%t(Z[,,i])%*%I[i,],warning=function(e) F,error=function(e) rep(0,ncol(Z)))}

Beta=matrix(0,nrow=dim(Z)[2],ncol=nrow(B))

for(i in 1:dim(Z)[2]){Beta[i,]=cumsum(beta[i,])}

Lambdaage=matrix(0,nrow=nrow(B),ncol=nrow(B))

for (i in 1:nrow(B)){for (j in 1:i){

Lambdaage[i,j]=Beta[1,j]+Beta[2,j]*B$age[i]}}

for(i in 1:(nrow(B)-1)){for(j in (i+1):nrow(B)){Lambdaage[i,j]=Lambdaage[i,i]}}

I=diag(as.numeric(B$status!=0))

Z=rep(0,nrow(B)*3*nrow(B))

dim(Z)=c(nrow(B),3,nrow(B))

Z[,1,]=1

Z[,2,]=exp(B$age/10)

Z[,3,]=exp((B$age-70)/10)*(B$age>70)

for (i in 2:nrow(B)){

Z[1:i-1,,i]=0}

beta=matrix(0,nrow=dim(Z)[2],ncol=nrow(B))

for (i in 1:nrow(B)){

beta[,i]=tryCatch(solve(crossprod(Z[,,i],Z[,,i]))%*%t(Z[,,i])%*%I[i,],warning=function(e) F,error=function(e) rep(0,ncol(Z)))}

Beta=matrix(0,nrow=dim(Z)[2],ncol=nrow(B))

for(i in 1:dim(Z)[2]){Beta[i,]=cumsum(beta[i,])}

Lambdaagee=matrix(0,nrow=nrow(B),ncol=nrow(B))

for (i in 1:nrow(B)){for (j in 1:i){

Lambdaagee[i,j]=Beta[1,j]+Beta[2,j]*exp(B$age[i]/10)+Beta[3,j]*exp((B$age[i]-70)/10)*(B$age[i]>70)}}

for(i in 1:(nrow(B)-1)){for(j in (i+1):nrow(B)){Lambdaagee[i,j]=Lambdaagee[i,i]}}

x11();par(mfrow=c(1,2))

B$agecat=cut(B$age,breaks=c(20,59.61,68.23,75.39,100))

plot(colSums(Lambdaage[which(B$agecat=="(20,59.6]"),])[B$agecat=="(20,59.6]"&B$status2==1]~cumsum(summary(survfit(Surv(time,status2)~1,data=B,subset=(agecat=="(20,59.6]")))$n.event),type="l",xlim=c(0,400),ylim=c(0,400),lty=2,lwd=5,col="grey0",xlab="Number of observed events",ylab="Number of estimated events",cex.lab=1.7,,cex.axis=1.7)

lines(colSums(Lambdaage[which(B$agecat=="(59.6,68.2]"),])[B$agecat=="(59.6,68.2]"&B$status2==1]~cumsum(summary(survfit(Surv(time,status2)~1,data=B,subset=(agecat=="(59.6,68.2]")))$n.event),type="l",lty=2,lwd=5,col="grey30")

lines(colSums(Lambdaage[which(B$agecat=="(68.2,75.4]"),])[B$agecat=="(68.2,75.4]"&B$status2==1]~cumsum(summary(survfit(Surv(time,status2)~1,data=B,subset=(agecat=="(68.2,75.4]")))$n.event),type="l",lty=2,lwd=5,col="grey60")

lines(colSums(Lambdaage[which(B$agecat=="(75.4,100]"),])[B$agecat=="(75.4,100]"&B$status2==1]~cumsum(summary(survfit(Surv(time,status2)~1,data=B,subset=(agecat=="(75.4,100]")))$n.event),type="l",lty=2,lwd=5,col="grey90")

abline(0,1)

legend(0,400,c(expression("age"<="59.6 years"),"age=(59.6,68.2] years","age=(68.2,75.4] years","age>75.4 years"),lty=2,lwd=5,col=c("grey0","grey30","grey60","grey90"),cex=1.7)

mtext("a",side=1,adj=0,line=3.5,cex=2.5)

plot(colSums(Lambdaagee[which(B$agecat=="(20,59.6]"),])[B$agecat=="(20,59.6]"&B$status2==1]~cumsum(summary(survfit(Surv(time,status2)~1,data=B,subset=(agecat=="(20,59.6]")))$n.event),type="l",xlim=c(0,400),ylim=c(0,400),lty=2,lwd=5,col="grey0",xlab="Number of observed events",ylab="Number of estimated events",cex.lab=1.7,cex.axis=1.7)

lines(colSums(Lambdaagee[which(B$agecat=="(59.6,68.2]"),])[B$agecat=="(59.6,68.2]"&B$status2==1]~cumsum(summary(survfit(Surv(time,status2)~1,data=B,subset=(agecat=="(59.6,68.2]")))$n.event),type="l",lty=2,lwd=5,col="grey30")

lines(colSums(Lambdaagee[which(B$agecat=="(68.2,75.4]"),])[B$agecat=="(68.2,75.4]"&B$status2==1]~cumsum(summary(survfit(Surv(time,status2)~1,data=B,subset=(agecat=="(68.2,75.4]")))$n.event),type="l",lty=2,lwd=5,col="grey60")

lines(colSums(Lambdaagee[which(B$agecat=="(75.4,100]"),])[B$agecat=="(75.4,100]"&B$status2==1]~cumsum(summary(survfit(Surv(time,status2)~1,data=B,subset=(agecat=="(75.4,100]")))$n.event),type="l",lty=2,lwd=5,col="grey90")

abline(0,1)

legend(0,400,c(expression("age"<="59.6 years"),"age=(59.6,68.2] years","age=(68.2,75.4] years","age>75.4 years"),lty=2,lwd=5,col=c("grey0","grey30","grey60","grey90"),cex=1.7)

mtext("b",side=1,adj=0,line=3.5,cex=2.5)

multivariate Aalen’s and Lin’s models

multAm=aareg(Surv(time,status!=0)~I(exp(age/10))+I(exp((age/10-7))*(age>70))+sex+chf+dia+vf,data=B)

summary(multAm)

multLinm=ahaz(Surv(B$time,B$status!=0),as.matrix(cbind(exp(B$age/10),exp((B$age/10-7))*(B$age>70),B$sex,B$chf,B$dia,B$vf)))

summary(multLinm)

# Martingale residual processes

#------------------------------

# Table 4 (Tests of martingale residual processes)

MRP(event=B$status2,covariates=as.matrix(cbind(exp(B$age/10),exp((B$age/10-7))*(B$age>70),B$sex,B$chf,B$dia,B$vf)),contcat=B$age2)

# Figure 12 (plots of cumulative hazards)

#~~~~~~~~~~~~~~~~~~~~~~~~~~~~~~~~~~~~~~~~

x11();par(mfrow=c(2,3))

plot(multAm[2],cex.axis=2,cex.main=3,main="exp(age/10)",col.lab="white")

abline(0,summary(multLinm)$coefficients[1,1],col=1,lty=2)

mtext("a",side=1,adj=0,line=3.5,cex=2.5)

mtext("Time (years)",side=1,adj=0.5,line=3.5,cex=1.7)

mtext("Beta(t)",side=2,adj=0.5,line=2.2,cex=1.7)

plot(multAm[3],cex.axis=2,cex.main=3,main="exp(age/10-7)*(age>70)",col.lab="white")

abline(0,summary(multLinm)$coefficients[2,1],col=1,lty=2)

mtext("b",side=1,adj=0,line=3.5,cex=2.5)

mtext("Time (years)",side=1,adj=0.5,line=3.5,cex=1.7)

mtext("Beta(t)",side=2,adj=0.5,line=2.5,cex=1.7)

plot(multAm[4],cex.axis=2,cex.main=3,main="sex",col.lab="white",ylim=c(0,0.8))

abline(0,summary(multLinm)$coefficients[3,1],col=1,lty=2)

mtext("c",side=1,adj=0,line=3.5,cex=2.5)

mtext("Time (years)",side=1,adj=0.5,line=3.5,cex=1.7)

mtext("Beta(t)",side=2,adj=0.5,line=2.5,cex=1.7)

plot(multAm[5],cex.axis=2,cex.main=3,main="chf",col.lab="white",ylim=c(0,0.8))

abline(0,summary(multLinm)$coefficients[4,1],col=1,lty=2)

mtext("d",side=1,adj=0,line=3.5,cex=2.5)

mtext("Time (years)",side=1,adj=0.5,line=3.5,cex=1.7)

mtext("Beta(t)",side=2,adj=0.5,line=2.2,cex=1.7)

plot(multAm[6],cex.axis=2,cex.main=3,main="dia",col.lab="white",ylim=c(0,0.8))

abline(0,summary(multLinm)$coefficients[5,1],col=1,lty=2)

mtext("e",side=1,adj=0,line=3.5,cex=2.5)

mtext("Time (years)",side=1,adj=0.5,line=3.5,cex=1.7)

mtext("Beta(t)",side=2,adj=0.5,line=2.5,cex=1.7)

plot(multAm[7],cex.axis=2,cex.main=3,main="vf",col.lab="white",ylim=c(0,0.8))

abline(0,summary(multLinm)$coefficients[6,1],col=1,lty=2)

mtext("f",side=1,adj=0,line=3.5,cex=2.5)

mtext("Time (years)",side=1,adj=0.5,line=3.5,cex=1.7)

mtext("Beta(t)",side=2,adj=0.5,line=2.5,cex=1.7)

# Figure 13 (cumulative hazards for age)

#~~~~~~~~~~~~~~~~~~~~~~~~~~~~~~~~~~~~~

x11()

j=function(x){(summary(multLinm)$coefficients[1,1]*exp(x/10)+summary(multLinm)$coefficients[2,1]*exp(x/10-7)*(x>70))}

jinf=function(x){(summary(multLinm)$coefficients[1,1]-1.96*summary(multLinm)$coefficients[1,2])*exp(x/10)+(summary(multLinm)$coefficients[2,1]-1.96*summary(multLinm)$coefficients[2,2])*exp(x/10-7)*(x>70)}

jsup=function(x){(summary(multLinm)$coefficients[1,1]+1.96*summary(multLinm)$coefficients[1,2])*exp(x/10)+(summary(multLinm)$coefficients[2,1]+1.96*summary(multLinm)$coefficients[2,2])*exp(x/10-7)*(x>70)}

curve(j,23,96,ylim=c(0,2),cex.axis=1.7,lwd=3,cex.lab=1.7,xlab="age (years)",ylab="Beta (t)",cex.main=2,main="")

curve(jinf,add=T,lty=2,lwd=3)

curve(jsup,add=T,lty=2,lwd=3)

abline(h=1,lty=3)
